# DKFraudNet: a knowledge-guided adversarial learning framework for fraud user detection

**DOI:** 10.3389/fdata.2026.1887075

**Published:** 2026-08-12

**Authors:** Yingjun Shen, Renda Shi, Kaixi Song, Yunpeng Li

**Affiliations:** 1 Shenzhen Finance Institute, School of Management and Economics, The Chinese University of Hong Kong, Shenzhen, Guangdong, China; 2 School of Economics and Management, Southeast University, Nanjing, Jiangsu, China; 3 School of Data Science, The Chinese University of Hong Kong, Shenzhen, Guangdong, China

**Keywords:** ambiguous sample refinement, domain knowledge, fraud detection, generative adversarial networks, weak supervision

## Abstract

**Introduction:**

Fraud user identification in telecommunications is hindered by scarce, noisy, and imbalanced labels, while expert rules may provide ambiguous or contradictory evidence.

**Methods:**

We propose DKFraudNet, a knowledge-guided framework that integrates domain knowledge regularization, an attention-adaptive conditional generative adversarial network, and virtual category learning. Expert rules are organized into a Deterministic-Ambiguous-Contradictory evidence taxonomy for controlled pseudo-labeling. Class-conditioned augmentation alleviates data imbalance, and kernel-based similarity refines ambiguous samples. The framework was evaluated using two real-world city-level telecommunications datasets containing 59,015 and 52,183 users, respectively.

**Results:**

DKFraudNet consistently improved downstream classification performance under weak supervision. On the independent City 2 validation set, the CatBoost instantiation achieved an accuracy of 0.911 and an F1-score of 0.909. For operational review prioritization, DKFraudNet-XGBoost required reviewing 40.2% of unverified users to capture 80% of fraud users, corresponding to a 49.71% workload reduction relative to random review, with an AUPRC of 0.954. In operator-side blind verification, genuine-member identification and false-member screening achieved accuracies of 98.9% and 93.3%, respectively.

**Discussion:**

The results show that combining structured domain evidence, adaptive generative augmentation, and uncertainty-aware refinement improves robustness, data efficiency, and operational usefulness for fraud detection under weak supervision.

## 1 Introduction

Non-genuine and fraudulent users pose operational risk in large-scale digital service systems, particularly in telecommunications, where user identities, corporate affiliations, service subscriptions, billing records, communication behaviors, and location-related attributes are continuously updated. Within corporate customer groups, erroneous or manipulated affiliation records may introduce illegitimate users into valid enterprise accounts, thereby distorting customer profiles, undermining relationship management, and causing economic losses (Li and Zhang, 2021; Mort and Drennan, 2005). As digital services become increasingly interconnected, fraudulent behaviors may evolve rapidly, resulting in asymmetry between adaptive attacks and relatively static detection systems (Rathore et al., 2021; Alves Gomes and Meisen, 2023).

Even fraud detection has been extensively studied, reliable deployment in real-world telecommunications systems remains challenging. The first challenge is label uncertainty. High-quality labels are difficult to obtain because manual verification is costly, time-consuming, privacy-sensitive, and often dependent on inconsistent human judgment (Bruni and Bianchi, 2020; Frenay and Verleysen, 2014). Operational datasets may contain missing or noisy labels. Recent studies emphasize that data quality issues, including noise, incompleteness, and inconsistent annotation, can significantly affect the reliability of fraud detection models (Patel, 2023; Gouthier et al., 2022). The second challenge is class imbalance. Fraudulent or illegitimate users usually account for a small fraction of the entire population, causing standard classifiers to favor the majority class and reducing sensitivity to rare suspicious cases (Madkour et al., 2024). The third challenge is concept drift and adversarial adaptation. Fraudsters may adjust their behavior to imitate legitimate users or exploit changing operational rules, which weakens the generalization ability of models trained on historical data (Abdallah et al., 2016).

Rule-based systems are interpretable and efficient, but they rely heavily on manually designed thresholds and require continuous maintenance as fraud strategies evolve. Statistical and classical machine learning methods can capture empirical regularities, but their performance is often degraded by sparse signals and distribution shifts (Sharma et al., 2022; Alkhayrat et al., 2020). Unsupervised and semi-supervised methods reduce dependence on labeled data, but they may produce high false positive rates or provide insufficient interpretability in high-dimensional data. In practice, fraud detection requires not only accurate prediction but also business plausibility, controllable uncertainty, and operational interpretability.

This paper proposes DKFraudNet, a knowledge-guided framework for robust fraud user identification in telecommunications systems. The framework integrates three complementary components. First, domain knowledge regularization (DKR) formalizes expert rules into a Deterministic–Ambiguous–Contradictory (DAC) taxonomy and uses rule-evidence scores to generate high-confidence pseudo-labels while explicitly identifying ambiguous and conflicting cases. Second, an attention-adaptive conditional generative adversarial network (AAC-GAN) synthesizes user profiles conditioned on class information and selected discriminative features, thereby alleviating class imbalance under limited supervision. Third, a virtual category learning mechanism preserves uncertain samples as an intermediate category and refines their final assignments through kernel-based similarity to class prototypes. By integrating these components, DKFraudNet aims to improve robustness, interpretability, and data efficiency under noisy, imbalanced, and weakly supervised conditions.

The main contributions of this study are summarized as follows:

- **Knowledge-guided pseudo-labeling under weak supervision**. We propose a domain knowledge regularization mechanism that organizes expert rules into a Deterministic–Ambiguous–Contradictory taxonomy. This design transforms business logic into structured rule evidence and enables high-confidence pseudo-label generation while preserving ambiguous and conflicting samples for further refinement.
- **Attention-adaptive generative augmentation for imbalanced data**. We develop an attention-adaptive conditional GAN that synthesizes user profiles by conditioning generation on class labels and discriminative behavioral features. The attention mechanism emphasizes informative feature dimensions, improving the utility of generated samples for fraud detection.
- **Virtual category refinement for uncertain samples**. We introduce a virtual category learning mechanism to explicitly represent samples with uncertain or insufficient evidence, which refines them through kernel-based similarity to class centroids.
- **Real-world validation on telecommunications data**. We evaluate DKFraudNet using real-world user-level and group-level data from a major telecommunications operator. The experiments compare the proposed framework's effectiveness under operational data constraints.

The remainder of this paper is organized as follows. Section 2 reviews related studies on fraud detection. Section 3 formulates the fraud user identification problem. Section 4 presents the proposed DKFraudNet framework. Section 5 reports the experimental results and analysis. Finally, Section 6 concludes the paper and discusses future research directions.

## 2 Related work

This section positions DKFraudNet within the literature on fraud detection. The proposed framework targets telecommunications fraud user identification under scarce, noisy, and imbalanced labels. Accordingly, we review related studies from five perspectives: fraud detection in telecommunications and digital service systems, traditional and learning-based fraud detection methods, knowledge-guided and weakly supervised detection, generative augmentation for imbalanced fraud detection, and uncertainty-aware learning.

### 2.1 Fraud detection in telecommunications and digital service systems

Fraud detection has been studied across telecommunications, finance, insurance, e-commerce, and online service platforms (Hu et al., 2011; Asha and Suresh Kumar, 2021). In telecommunications systems, fraudulent behavior may involve identity misuse, abnormal account affiliation, illegitimate group membership, account takeover, collusive activity, and synthetic usage patterns designed to appear legitimate (Liu et al., 2020). Telecommunications fraud user identification often requires integrating heterogeneous information, including customer registration, tariff plans, group affiliation, payment records, communication behavior, and spatiotemporal attributes.

A key difficulty is that fraudulent behavior can be subtle, adaptive, and context-dependent. Suspicious users may not exhibit extreme anomalies in a single variable, but may deviate from legitimate users through combinations of weak signals. For instance, a user may appear normal in terms of payment or subscription plan but display inconsistent group affiliation, weak interaction with group members, or abnormal geographic patterns. At the same time, legitimate users may also show rare or irregular behavior, making simple threshold-based detection prone to false positives. These characteristics require detection models that can capture complex feature interactions while remaining interpretable for operational use.

Real-world fraud datasets are often incomplete, noisy, and weakly labeled, and fraud patterns can change over time. Concept drift, adversarial adaptation, and changing business rules reduce the reliability of models trained on historical data (Anna et al., 2021). Therefore, effective telecommunications fraud detection requires not only predictive accuracy but also robustness to imperfect labels, imbalanced class distributions, and evolving behavioral patterns.

### 2.2 Traditional, hybrid, and ensemble fraud detection methods

Traditional fraud detection methods can be broadly grouped into rule-based systems, statistical anomaly detection, classical machine learning, and hybrid or ensemble learning methods. Rule-based systems are the most widely deployed approaches. They encode expert knowledge using deterministic conditions, such as threshold rules or if-then logic, and are valued for their interpretability, auditability, and real-time applicability (Fawcett and Provost, 1997; Kou et al., 2004). In telecommunications fraud detection, rule-based methods have been used to capture known suspicious patterns and support operational decision-making (Hilas, 2009). However, they depend heavily on manual rule engineering. As fraud strategies evolve, fixed rules may become outdated, and maintaining large rule sets can be labor-intensive and error-prone. Moreover, rigid rules often have limited ability to detect novel, collusive, or context-dependent fraud patterns (Sanchez et al., 2009).

Statistical anomaly detection methods treat fraud as a deviation from normal behavior. Common techniques include density estimation, hypothesis testing, clustering, and outlier detection (Singh and Upadhyaya, 2012; Blázquez-García et al., 2021). These methods are useful when fraudulent behavior produces clear statistical deviations. However, in telecommunications scenarios, fraud may be deliberately designed to resemble legitimate behavior, while rare legitimate users may also appear anomalous. As a result, statistical methods can suffer from high false positive rates when the distinction between rare normal behavior and malicious behavior is ambiguous.

Classical machine learning methods learn predictive patterns from historical data and have become a major paradigm in fraud detection. Supervised models such as decision trees, Bayesian networks, and adaptive classifiers have been applied to fraud classification tasks (Sahin et al., 2013; Song et al., 2020; Pranto et al., 2022). Unsupervised methods, including clustering and anomaly detection, are useful when labels are unavailable or unreliable (Hilas and Mastorocostas, 2008). Semi-supervised learning further exploits limited labeled data together with abundant unlabeled data to improve generalization (Dzakiyullah et al., 2021). Nevertheless, these methods may degrade when labels are noisy, minority fraud cases are rare, or fraud patterns shift over time.

To improve robustness and predictive performance, hybrid and ensemble approaches have also been widely studied. Ensemble learning combines multiple base learners through strategies such as bagging, boosting, and stacking, and is effective in high-dimensional and imbalanced settings (Xu et al., 2023; Louzada and Ara, 2012). Fuzzy-logic-based hybrid systems incorporate rule-based reasoning to support fraud detection under uncertainty (Charizanos et al., 2024; Faisal et al., 2024), while hybrid models combining unsupervised outlier detection with supervised classification exploit both anomaly signals and labeled fraud patterns (Carcillo et al., 2021). These approaches can improve empirical performance by reducing variance, capturing nonlinear patterns, and integrating complementary information sources.

Despite these advances, traditional, hybrid, and ensemble methods still face important limitations in real-world fraud detection. Many of them assume that training data are representative of future operational data, which may not hold when attackers adapt their strategies, business rules change, or labels are delayed and corrected after deployment. In addition, complex ensembles may reduce interpretability, which is problematic in operational environments where decisions need to be explained to business analysts or risk-control teams (Yang et al., 2021).

### 2.3 Knowledge-guided and weakly supervised fraud detection

Domain knowledge remains essential in operational fraud detection because business experts often understand fraud patterns that are difficult to infer from limited labeled data alone. Expert knowledge may describe abnormal group affiliation, inconsistent tariff usage, unusual payment behavior, weak interaction with legitimate members, or suspicious geographic patterns. Prior work has recognized the value of human expertise and domain-specific knowledge in identifying abnormal or deceptive behavior (Šubelj et al., 2011). Such knowledge can guide feature construction, constrain model predictions, support pseudo-labeling, and improve interpretability. In weakly supervised settings, such knowledge can also be used to generate pseudo-labels or weak labels when verified labels are scarce.

Recent studies have investigated different ways to integrate knowledge with machine learning. Belief rule-based systems use structured rules and belief degrees to support reasoning under uncertainty (Hossain et al., 2022). Hybrid intelligent systems combine rule-based reasoning with data-driven models to improve decision support (Peddabachigari et al., 2007). Knowledge-guided neural networks incorporate expert constraints or domain priors into neural learning processes (Yang and Zhu, 2024). These approaches indicate that domain knowledge can help reduce dependence on large labeled datasets and improve the transparency of model behavior.

Nevertheless, many existing knowledge-guided methods treat expert rules as fixed constraints or deterministic labels. This is limiting in fraud detection because domain rules may be incomplete, context-dependent, or mutually inconsistent. For example, one rule may indicate that a user is likely legitimate based on payment stability, while another may indicate suspicious behavior due to weak group interaction. If such conflicts are ignored, rule-based pseudo-labels may propagate errors into downstream models. Therefore, knowledge-guided fraud detection should distinguish reliable, ambiguous, and contradictory evidence rather than forcing all rule outputs into hard labels.

### 2.4 Generative data augmentation for imbalanced fraud detection

Class imbalance is a fundamental challenge in fraud detection because fraudulent or illegitimate cases are usually rare relative to normal cases. Standard classifiers trained on imbalanced data tend to favor the majority class, resulting in low recall for minority suspicious users. Traditional imbalance-learning techniques include random oversampling, undersampling, cost-sensitive learning, and synthetic minority oversampling methods such as SMOTE (Ileberi et al., 2021; Alamri and Ykhlef, 2022). These techniques can improve class balance, but they may also introduce drawbacks. Oversampling can increase overfitting, undersampling may discard useful majority-class information, and interpolation-based synthetic samples may distort the original data geometry or amplify noise.

In addition to resampling, recommendation-based and context-aware modeling strategies have been explored to improve detection in sparse or multi-context environments. Collaborative filtering methods can exploit similarity patterns among users or behaviors (Ren et al., 2023), while multi-context approaches such as Ranking Metric Embedding-Based Multicontextual Behavior (ReMEMBeR) incorporate contextual information to improve representation and detection under sparse observations (Cui et al., 2021). These methods highlight the importance of using additional structure beyond individual feature vectors. However, they often depend on the availability and quality of auxiliary relational or contextual data, which may not always be accessible in operational telecommunications systems.

Generative models provide another way to address imbalance by learning the data distribution and synthesizing additional samples. GAN-based methods have been increasingly applied to fraud detection and imbalanced classification because they can generate synthetic minority-class samples when properly trained (Strelcenia and Prakoonwit, 2023; Yu et al., 2020). Conditional GANs further allow generation under class-specific conditions, making them suitable for controlled augmentation in supervised or weakly supervised settings (Hamghalam and Simpson, 2024). However, GANs are difficult to train on tabular, heterogeneous, noisy, and highly imbalanced operational data. They may suffer from mode collapse, instability, and sensitivity to hyperparameters. More importantly, generated samples must be not only statistically similar to real samples but also semantically plausible under business logic. Existing GAN-based methods often lack explicit domain guidance or feature-level adaptivity. The proposed AAC-GAN addresses this limitation by using attention-adaptive conditioning to emphasize discriminative features during generation.

### 2.5 Learning under label uncertainty and ambiguous sample refinement

Label uncertainty is common in real-world fraud detection. Labels may be generated from manual review, delayed investigation, heuristic rules, or partially verified operational records. Such labels can be noisy because of human error, inconsistent standards, incomplete evidence, or strategic behavior by fraudsters (Herland et al., 2018; Brahimi and Elhussein, 2023). Training models directly on unreliable labels can bias decision boundaries and reduce generalization, especially when the labeled set is small and the minority class is rare.

Several approaches have been proposed to handle uncertain or noisy labels. Noise-robust learning modifies training objectives to reduce sensitivity to mislabeled samples. Semi-supervised learning uses unlabeled data together with limited labeled data to improve representation learning and decision boundaries (Dzakiyullah et al., 2021). Soft labeling assigns probabilistic labels rather than hard binary labels to uncertain samples (Wang et al., 2023). Pseudo-labeling expands the training set by assigning labels to high-confidence unlabeled instances. Virtual or auxiliary category methods introduce additional categories to represent ambiguous samples instead of forcing every instance into predefined binary classes (Ma et al., 2019).

Despite these advances, uncertainty handling remains difficult in fraud detection. Confidence thresholds used in pseudo-labeling or soft labeling are hard to generalize. Ambiguous samples may be discarded, down-weighted, or assigned uncertain probabilities without further structural refinement. However, such samples are often informative because they lie near decision boundaries or represent atypical but operationally important user behavior. A more structured strategy is to preserve ambiguous samples as an intermediate category and refine them using reliable class structure. DKFraudNet follows this principle by introducing a virtual category and applying kernel-based centroid similarity to reassign uncertain samples after feature selection and generative augmentation.

### 2.6 Research gaps

The reviewed literature indicates three important gaps. First, although domain knowledge is widely used in fraud detection, existing methods often encode it as fixed rules, auxiliary variables, or deterministic constraints. They rarely distinguish deterministic, ambiguous, and contradictory rule evidence, which can lead to unreliable pseudo-labels under weak supervision. Second, generative augmentation methods can mitigate class imbalance, but most GAN-based approaches lack explicit domain guidance and feature-level adaptivity. As a result, generated samples may be statistically plausible but operationally unrealistic. Third, label uncertainty is often handled through heuristic filtering, soft labels, or confidence thresholds, while ambiguous samples are seldom modeled as a structured intermediate category with subsequent refinement.

To address these gaps, DKFraudNet integrates domain knowledge regularization, attention-adaptive conditional generation, and virtual category refinement into a unified framework. The proposed approach uses expert rules to generate controlled pseudo-labels, employs adaptive generative augmentation to alleviate imbalance, and refines uncertain samples through kernel-based similarity. This design is intended to improve fraud user identification under the noisy, imbalanced, and weakly supervised conditions commonly observed in real-world telecommunications data.

## 3 Problem formulation for fraud user identification

We consider the problem of identifying non-genuine users in large-scale telecommunications corporate. In the operational database, all users are initially recorded as members of corporate groups. However,some users may be non-genuine members due to erroneous affiliation records, outdated registration information, identity misuse, or other operational inconsistencies.

Each user is represented by a feature vector ${\text{x}}_{i}\in {\mathbb{R}}^{d}$, where the features may include demographic attributes, tariff information, group affiliation records, communication behavior, payment activities, temporal records, and location-related signals. Following the labeling convention, the true label is denoted by *y*_*i*_ ∈ {0, 1}, where *y*_*i*_ = 0 indicates a genuine group member and *y*_*i*_ = 1 indicates a non-genuine or illegitimate member. In practice, however, the true label *y*_*i*_ is not fully observable. Only a small subset of users has verified labels obtained from reliable business verification, while most users remain unverified. We denote an observed but potentially unreliable operational label by ỹ_*i*_ and a pseudo-label generated by the proposed method by ŷ_*i*_.

**Figure 1:**
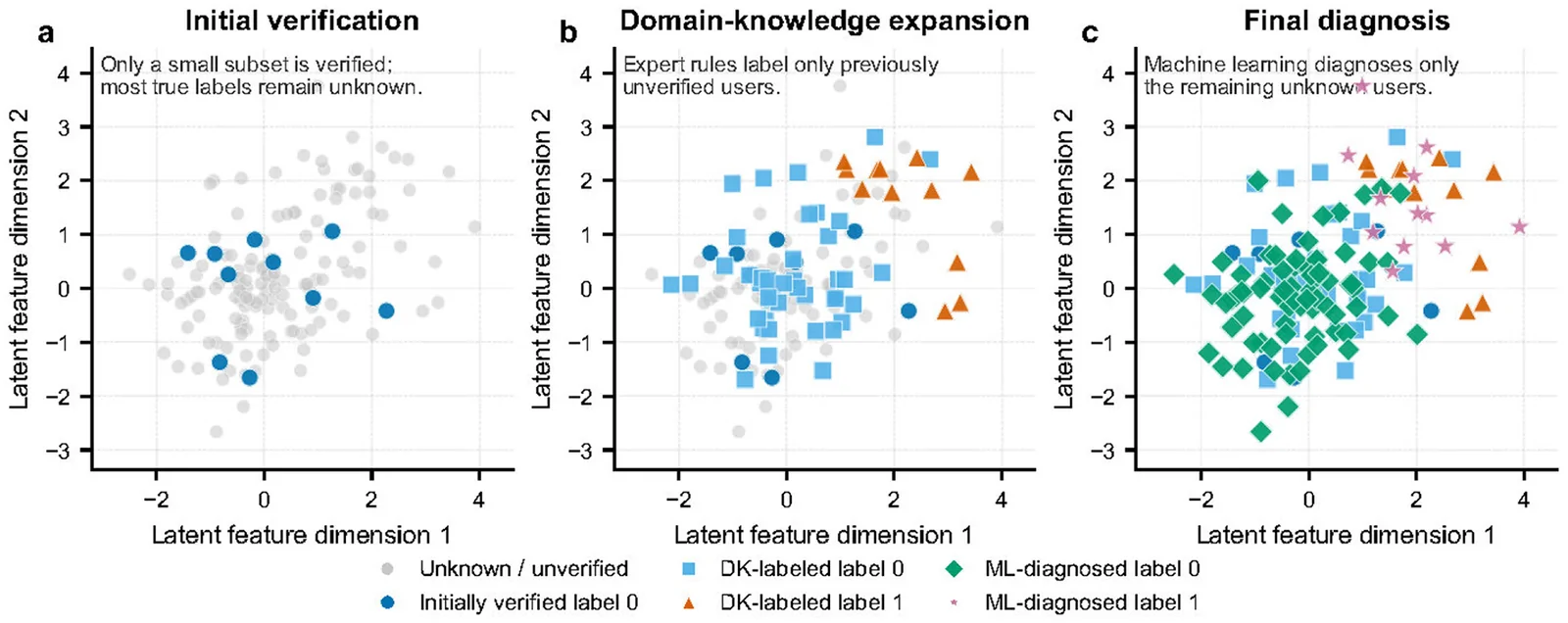
Illustration of the cumulative label-expansion process for fraud user identification. All users are initially recorded as corporate group users, but only a small subset has verified true labels. Domain knowledge assigns reliable labels to part of the previously unverified users, and machine learning further diagnoses the remaining unknown users. Label 0 denotes a genuine group member, whereas label 1 denotes a non-genuine or illegitimate member. **(a)** Initial verification, **(b)** Domain-knowlwedge expansion, and **(c)** final diagnosis.

Figure 1 illustrates the cumulative label-expansion process considered in this study. In the initial verification stage, only a few users are confirmed as genuine group members, whereas most users remain unverified. Domain knowledge is then used to assign reliable labels to part of the previously unverified users according to expert rules and business logic. Finally, machine learning diagnoses the remaining unverified users, while labels obtained in earlier stages are preserved.

The available dataset is denoted by:


$$\begin{array}{l} D=D^{l}\cup D^{u}, \end{array}$$ (1)

where D^*l*^ is a small set of samples with verified labels:


$$\begin{array}{l} D^{l}={\left\{\left({\text{x}}_{i}^{l},y_{i}^{l}\right)\right\}}_{i=1}^{N_{l}}, \end{array}$$ (2)

and D^*u*^ is a large set of unverified samples whose true labels are unknown:


$$\begin{array}{l} D^{u}={\left\{{\text{x}}_{i}^{u}\right\}}_{i=1}^{N_{u}}. \end{array}$$ (3)

Here, *N*_*l*_≪*N*_*u*_ and *N* = *N*_*l*_+*N*_*u*_. For samples in D^*u*^, operational records may contain weak or noisy label information ỹ_*i*_, but such labels are not directly treated as reliable ground truth.

The objective is to learn a classifier:


$$\begin{array}{l} f_{\Theta }:{\mathbb{R}}^{d}\to \left\{0,1\right\}, \end{array}$$ (4)

that predicts reliable user categories for unverified users while reducing the impact of label uncertainty, class imbalance, and noisy domain evidence. Specifically, the classifier aims to distinguish genuine group members from non-genuine or illegitimate members:


$$\begin{array}{l} {ŷ}_{i}=f_{\Theta }\left({\text{x}}_{i}\right), \end{array}$$ (5)

where ŷ_*i*_ = 0 indicates a predicted genuine group member and ŷ_*i*_ = 1 indicates a predicted non-genuine member. The dataset definition and prediction objective are summarized in Equations 1–5.

To address this problem, DKFraudNet follows a cumulative label-expansion strategy. First, domain knowledge regularization generates controlled pseudo-labels for samples with sufficient rule-based evidence and identifies ambiguous samples. Second, attention-adaptive conditional generative augmentation mitigates class imbalance by enriching the training data. Third, virtual category learning refines uncertain samples before final classification. This formulation enables the model to exploit verified labels, domain knowledge, and machine learning in a unified‘framework.

## 4 DKFraudNet: a unified framework for knowledge-guided fraud detection

### 4.1 Framework overview

We propose DKFraudNet, a unified framework that integrates domain knowledge, generative augmentation, and uncertainty-aware learning. The framework follows the problem setting defined in Section 3, where label 0 denotes a genuine group member and label 1 denotes a non-genuine or illegitimate member.

**Figure 2:**
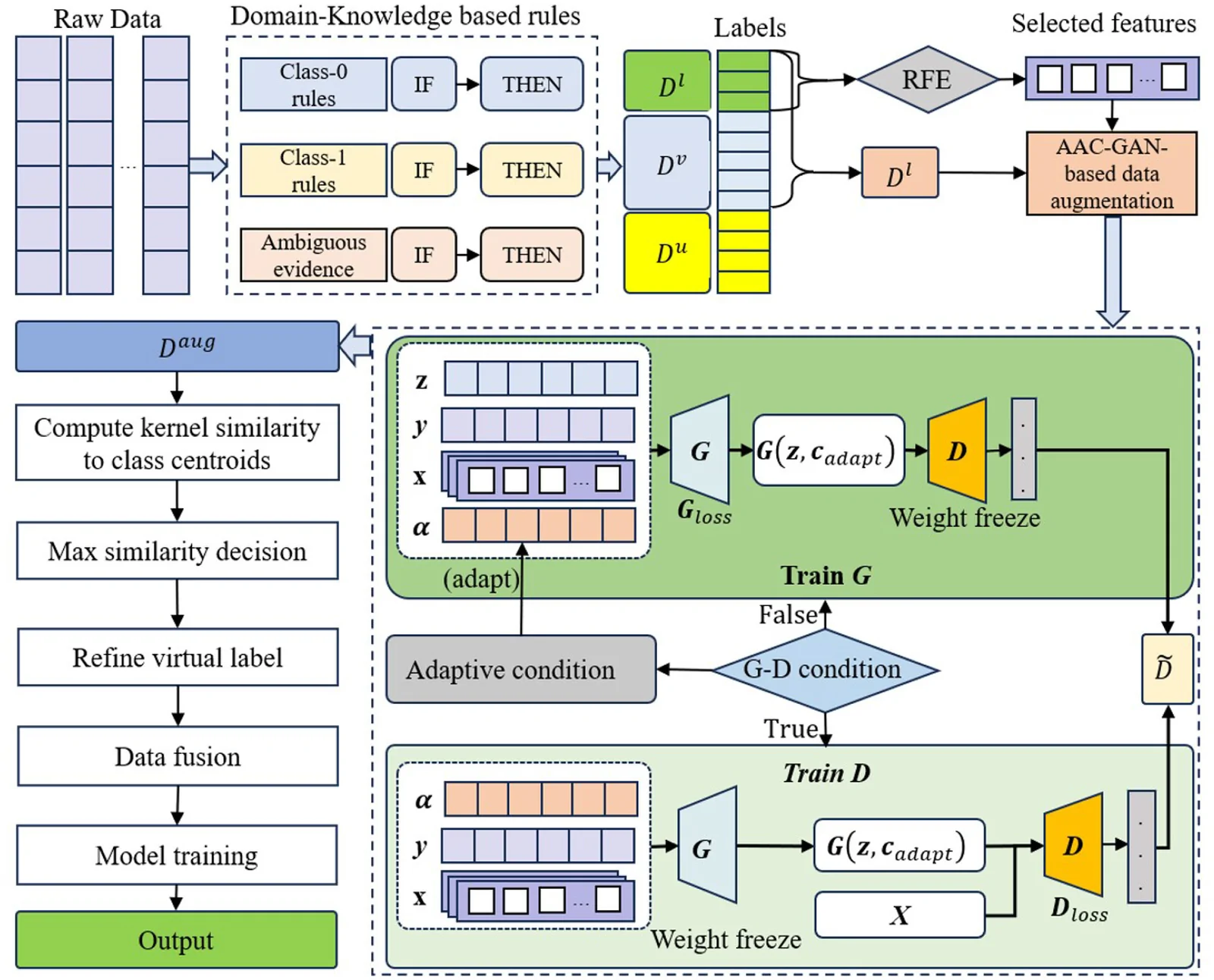
Overall framework of DKFraudNet.

As shown in Figure 2, DKFraudNet consists of four main stages. First, Domain Knowledge Regularization (DKR) converts expert rules into structured class-specific evidence and generates controlled pseudo-labels for samples with sufficient and non-conflicting evidence. Samples with ambiguous evidence are assigned to a virtual category for subsequent refinement, while samples with insufficient or contradictory evidence remain unknown. Second, feature construction and Recursive Feature Elimination (RFE) are performed to obtain an informative and interpretable feature subset. Third, an Attention-Adaptive Conditional GAN (AAC-GAN) generates synthetic samples conditioned on class labels and selected feature representations to alleviate class imbalance. Finally, Virtual Learning modeling (VLM) refines ambiguous samples through kernel-based similarity to class centroids, and the resulting augmented and refined dataset is used for final classifier training.

A key characteristic of DKFraudNet is its cumulative label-expansion strategy. Labels obtained in earlier stages are preserved, and later stages are applied only to samples that remain uncertain or unlabeled. The framework expands reliable supervision step by step by combining verified labels, domain knowledge, generative augmentation, and machine learning.

The complete training procedure is summarized in Algorithm 1.

Algorithm 1 Training Procedure of DKFraudNet. 
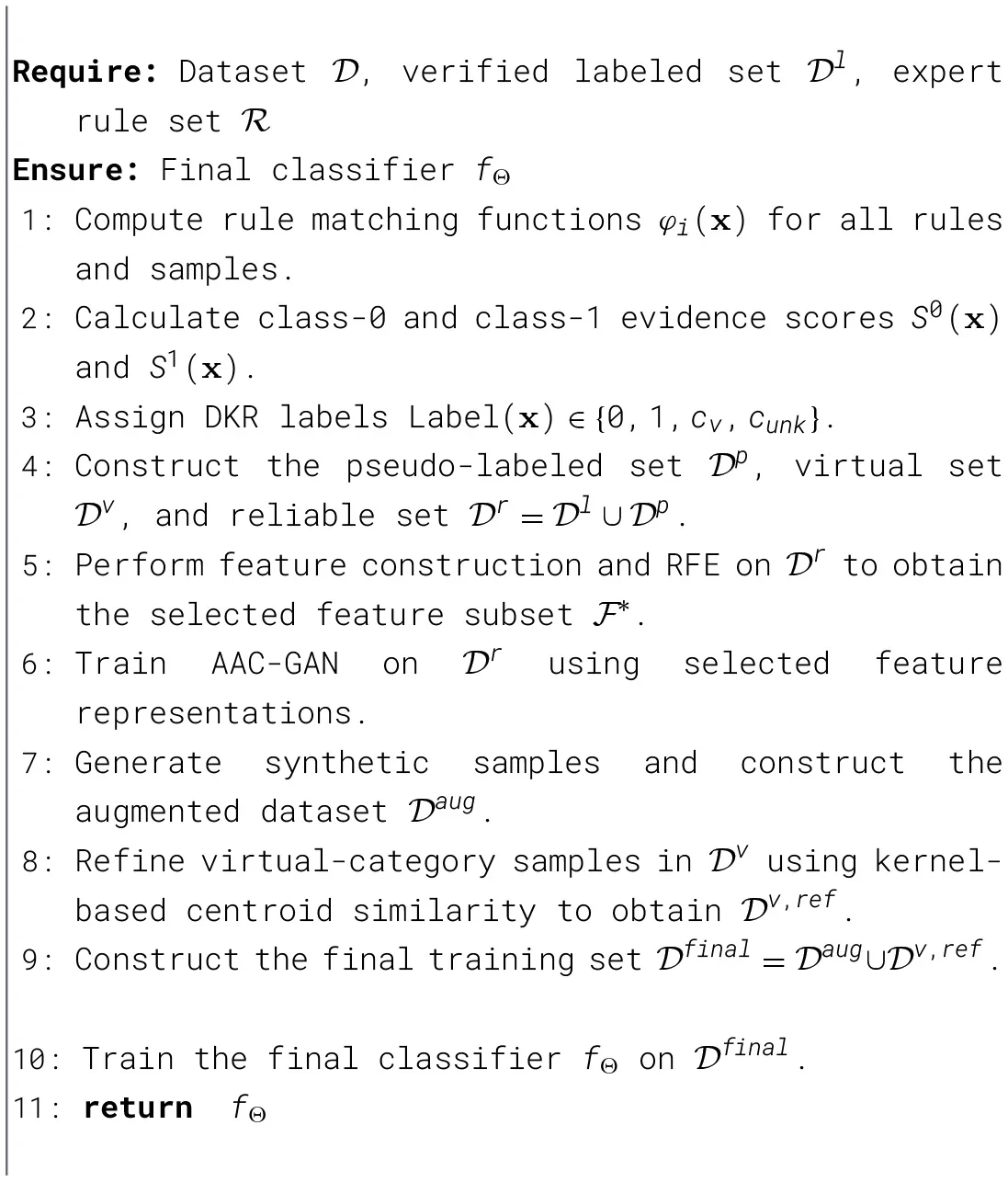

### 4.2 Domain knowledge regularization for pseudo-labeling

The DKR module transforms expert rules into structured evidence for weakly supervised pseudo-labeling. DKR distinguishes three types of evidence: deterministic evidence, ambiguous evidence, and contradictory evidence. Deterministic evidence provides reliable support for either the genuine-member class or the non-genuine-member class. Ambiguous evidence indicates that rule activation is informative but not strong enough for deterministic labeling. Contradictory evidence occurs when rules supporting different classes are simultaneously activated.

Let D = {*R*_1_, *R*_2_, …, *R*_*m*_} denote a set of expert-defined rules. Each rule *R*_*i*_ is formalized as a logical “if-then” expression and is associated with a binary matching function:


$$\begin{array}{l} \varphi_{i}:{\mathbb{R}}^{d}\to \left\{0,1\right\}, \end{array}$$ (6)

where, for any input feature vector **x** ∈ ℝ^*d*^, φ_*i*_(**x**) = 1 if rule *R*_*i*_ is satisfied and φ_*i*_(**x**) = 0 otherwise.

Following the label convention in this study, deterministic rules are divided into two groups:

- **Class-0 rules** (R^0^): rules that provide evidence that a user is a genuine group member.
- **Class-1 rules** (R^1^): rules that provide evidence that a user is a non-genuine or illegitimate member.

For a given sample **x**, the class-0 and class-1 deterministic evidence scores are computed by aggregating rule activations:


$$\begin{array}{l} S^{0}\left(\text{x}\right)=\overset{N^{0}}{\underset{i=1}{\sum }}\omega_{i}^{0}\varphi_{i}^{0}\left(\text{x}\right), \end{array}$$ (7)

and


$$\begin{array}{l} S^{1}\left(\text{x}\right)=\overset{N^{1}}{\underset{j=1}{\sum }}\omega_{j}^{1}\varphi_{j}^{1}\left(\text{x}\right), \end{array}$$ (8)

where $\omega_{i}^{0}$ and $\omega_{j}^{1}$ are rule weights, *N*^0^ and *N*^1^ denote the numbers of class-0 and class-1 rules, respectively, and $\varphi_{i}^{0}$ and $\varphi_{j}^{1}$ are the matching functions corresponding to rules in R^0^ and R^1^.

Contradictory evidence is detected when both classes receive nonzero rule support:


$$\begin{array}{l} C\left(\text{x}\right)=\text{𝕀}\left(S^{0}\left(\text{x}\right)>0\wedge S^{1}\left(\text{x}\right)>0\right), \end{array}$$ (9)

where *C*(**x**) = 1 indicates the existence of conflicting rule evidence.

Ambiguous evidence is identified when the rule evidence is neither strong enough for deterministic pseudo-labeling nor completely absent. We define the ambiguity indicator as


$$\begin{array}{l} \text{Φ}(x)=I(\left(S^{0}(x)\in [\theta_{l},\theta^{0})\wedge S^{1}(x)=0\right)\\ \vee (S^{0}(x)=0\wedge S^{1}(x)\in [\theta_{l},\theta^{1}))), \end{array}$$ (10)

where θ_*l*_ define the lower bound of the ambiguous evidence interval, θ^0^ and θ^1^ are deterministic evidence thresholds for label 0 and label 1, respectively. Φ(*x*) is also referred to as the virtual-category indicator and Φ(**x**) = 1 indicates that **x** is assigned to the virtual category for subsequent refinement. The rule-matching functions, class-specific evidence measures, and ambiguity indicator are defined in Equations 6–10.

We define four pseudo-labels, denoted by 0, 1, *c*_*v*_, and *c*_unk_. Specifically, 0 and 1 correspond to class-0 and class-1, respectively; *c*_*v*_ denotes the virtual category; and *c*_unk_ denotes samples with insufficient or contradictory evidence. Based on the evidence scores defined above, the DKR pseudo-labeling function is formulated as:


$$\mathrm{Label}(x)=\{\begin{array}{ll} 0, & \text{if }S^{0}(x)\geq \theta^{0}\text{ and }S^{1}(x)=0,\\ 1, & \text{if }S^{1}(x)\geq \theta^{1}\text{ and }S^{0}(x)=0,\\ c_{v}, & \text{if Φ}(x)=1,\\ c_{unk}, & \text{if }C(x)=1\text{ or }\max\{S^{0}(x),S^{1}(x)\}<\theta_{l}. \end{array}$$ (11)

**Observation (pseudo-label consistency)**. If all activated deterministic rules support the same class for a sample **x**, then DKR assigns a pseudo-label consistent with the rule evidence. Specifically, if *S*^0^(**x**)>0 and *S*^1^(**x**) = 0, the pseudo-label is assigned to label 0 when *S*^0^(**x**)≥θ^0^. Similarly, if *S*^1^(**x**)>0 and *S*^0^(**x**) = 0, the pseudo-label is assigned to label 1 when *S*^1^(**x**)≥θ^1^. This property ensures that DKR preserves deterministic rule agreement while preventing weak or conflicting evidence from being converted into hard abels.

By applying Equation 11 to the unverified samples, DKR partitions the data into three subsets. The pseudo-labeled set is defined as:


$$\begin{array}{l} D^{p}=\left\{\left({\text{x}}_{i},{ŷ}_{i}\right)|\mathrm{Label}\left({\text{x}}_{i}\right)={ŷ}_{i}\right\}, \end{array}$$ (12)

where ŷ_*i*_ ∈ {0, 1} denotes a DKR-generated pseudo-label. The virtual-category set is defined as:


$$\begin{array}{l} D^{v}=\left\{{\text{x}}_{i}|\mathrm{Label}\left({\text{x}}_{i}\right)=c_{v}\right\}, \end{array}$$ (13)

and the unknown set is defined as:


$$\begin{array}{l} D^{unk}=\left\{{\text{x}}_{i}|\mathrm{Label}\left({\text{x}}_{i}\right)=c_{unk}\right\}. \end{array}$$ (14)

The reliable training set used in subsequent stages is constructed by combining the initially verified labeled set and the DKR pseudo-labeled set:


$$\begin{array}{l} D^{r}=D^{l}\cup D^{p}. \end{array}$$ (15)

The pseudo-label assignment and construction of the reliable, virtual, and unknown subsets are specified in Equations 12–15.

### 4.3 Feature construction and selection

After DKR, feature construction and selection are performed to obtain an informative and interpretable representation for downstream learning. The initial feature set includes raw operational variables and derived variables, such as temporal intervals, behavioral interaction measures, tariff-related attributes, and location-based distance measures.

Let the initial feature set be denoted by:


$$\begin{array}{l} F^{\left(0\right)}=\left\{f_{1},f_{2},\ldots ,f_{m}\right\}, \end{array}$$ (16)

where *m* is the number of constructed features, which may differ from the dimensionality of the raw input variables. Feature selection is implemented via Recursive Feature Elimination (RFE) on the reliable training set D^*r*^.

At iteration *t*, the retained feature subset is denoted by F^(*t*)^⊆F^(0)^. A base classifier *g*(·) is trained using F^(*t*)^, and an evaluation metric, such as AUC, yields a validation score. An importance score *s*^(*t*)^(*f*) is computed for each feature *f* ∈ F^(*t*)^; in this study, feature importance is measured using Gini impurity reduction. The least important feature is removed according to:


$$\begin{array}{l} F^{\left(t+1\right)}=F^{\left(t\right)}\backslash \arg\underset{f\in F^{\left(t\right)}}{\min}s^{\left(t\right)}\left(f\right) \end{array}$$ (17)

This process stops when the validation performance decreases beyond a tolerance δ or when the number of retained features reaches a predefined minimum *k*_min_. The final selected feature subset is denoted by:


$$\begin{array}{l} F^{*}=\arg\underset{f\in F^{\left(T\right)}}{\max}s^{\left(T\right)}\left(f\right). \end{array}$$ (18)

where *T* denotes the total number of iterations.

For each sample **x**_*i*_, the selected feature representation is denoted by:


$$\begin{array}{l} {\text{x}}_{i}^{*}={\text{x}}_{i}\left[F^{*}\right]\in {\mathbb{R}}^{p}, \end{array}$$ (19)

where *p* = |F^*^| is the number of selected features. The feature construction and selection procedure is given in Equations 16–20.

### 4.4 AAC-GAN-based data augmentation

To address limited reliable labels and class imbalance, we adopt an Attention-Adaptive Conditional Generative Adversarial Network (AAC-GAN) to synthesize additional training samples. Unlike a standard conditional GAN that conditions generation only on class labels, AAC-GAN incorporates an attention-weighted selected feature representation into the condition vector. This allows the generator to focus on discriminative feature dimensions during sample synthesis.

Given a reliable sample $\left({\text{x}}_{i}^{*},y_{i}\right)\in D^{r}$, where ${\text{x}}_{i}^{*}\in {\mathbb{R}}^{p}$ and *y*_*i*_ ∈ {0, 1}, the initial condition vector is defined as


$$\begin{array}{l} {\text{c}}_{i}=\left[y_{i},{\text{x}}_{i}^{*}\right]. \end{array}$$ (20)

#### 4.4.1 Adaptive conditioning mechanism

The adaptive mechanism refines the conditioning information through an attention module. For each selected feature vector ${\text{x}}_{i}^{*}\in {\mathbb{R}}^{p}$, an intermediate representation is computed as:


$$\begin{array}{l} {\text{u}}_{i}=\tanh\left({\text{W}}_{a}{\text{x}}_{i}^{*}+{\text{b}}_{a}\right), \end{array}$$ (21)

where ${\text{W}}_{a}\in {\mathbb{R}}^{p\times p}$ is a learnable weight matrix and ${\text{b}}_{a}\in {\mathbb{R}}^{p}$ is a bias vector. A score vector is then obtained by:


$$\begin{array}{l} {\text{s}}_{i}={\text{W}}_{s}{\text{u}}_{i}+{\text{b}}_{s}, \end{array}$$ (22)

where ${\text{W}}_{s}\in {\mathbb{R}}^{p\times p}$ and ${\text{b}}_{s}\in {\mathbb{R}}^{p}$. The attention weights are computed using the softmax function:


$$\begin{array}{l} \alpha_{ij}=\frac{\exp\left(s_{ij}\right)}{\sum_{k=1}^{p}\exp\left(s_{ik}\right)},\;j=1,2,\ldots ,p. \end{array}$$ (23)

Let $\alpha_{i}={\left[\alpha_{i1},\ldots ,\alpha_{ip}\right]}^{⊤}$. The adaptive condition vector is defined as:


$$\begin{array}{l} {\text{c}}_{i}^{adapt}=\left[y_{i},\alpha_{i}⊙{\text{x}}_{i}^{*}\right], \end{array}$$ (24)

where ⊙ denotes element-wise multiplication.

#### 4.4.2 Generator

The generator *G* synthesizes feature representations that resemble real samples under the adaptive condition. Let **z** be a random noise vector sampled from a standard normal distribution:


$$\begin{array}{l} \text{z}\sim N\left(\text{0},\text{I}\right). \end{array}$$ (25)

Given **z** and the adaptive condition ${\text{c}}_{i}^{adapt}$, the generator produces a synthetic feature vector:


$$\begin{array}{l} {\tilde{\text{x}}}_{i}=G\left(\text{z},{\text{c}}_{i}^{adapt}\right). \end{array}$$ (26)

The label of the generated sample is inherited from the conditioning label *y*_*i*_ rather than generated independently.

The generator is optimized by minimizing:


$$\begin{array}{l} L_{G}=-{\text{𝔼}}_{\text{z}\sim N\left(\text{0},\text{I}\right)}\left[\log D\left(G\left(\text{z},{\text{c}}_{i}^{adapt}\right),{\text{c}}_{i}^{adapt}\right)\right], \end{array}$$ (27)

where $D\left(\cdot ,{\text{c}}_{i}^{adapt}\right)$ is the discriminator's estimated probability that the generated sample is real under the adaptive condition.

#### 4.4.3 Discriminator

The discriminator *D* distinguishes real samples from synthetic samples while being conditioned on ${\text{c}}_{i}^{adapt}$. Given a feature vector **x** and its adaptive condition, the discriminator outputs


$$\begin{array}{l} D\left(\text{x},{\text{c}}_{i}^{adapt}\right)\in \left[0,1\right], \end{array}$$ (28)

which represents the probability that **x** is real. The discriminator loss is defined as


$$\begin{array}{l} L_{D}=-{\text{𝔼}}_{\left({\text{x}}_{i}^{*},y_{i}\right)\sim D^{r}}\left[\log D\left({\text{x}}_{i}^{*},{\text{c}}_{i}^{adapt}\right)\right]\\ \;-{\text{𝔼}}_{\text{z}\sim N\left(\text{0},\text{I}\right)}\left[\log\left(1-D\left(G\left(\text{z},{\text{c}}_{i}^{adapt}\right),{\text{c}}_{i}^{adapt}\right)\right)\right]. \end{array}$$ (29)

#### 4.4.4 Adversarial objective

The overall adversarial training objective is formulated as:


$$\begin{array}{l} \underset{G}{\min}\underset{D}{\max}V\left(D,G\right), \end{array}$$ (30)

$$\begin{array}{l} V\left(D,G\right)={\text{𝔼}}_{\left({\text{x}}_{i}^{*},y_{i}\right)\sim D^{r}}\left[\log D\left({\text{x}}_{i}^{*},{\text{c}}_{i}^{adapt}\right)\right]\\ \;+{\text{𝔼}}_{\text{z}\sim N\left(\text{0},\text{I}\right)}\left[\log\left(1-D\left(G\left(\text{z},{\text{c}}_{i}^{adapt}\right),{\text{c}}_{i}^{adapt}\right)\right)\right]. \end{array}$$ (31)

Through this minimax game, the discriminator learns to distinguish real and synthetic samples, while the generator learns to synthesize realistic feature representations consistent with the adaptive condition. The adaptive conditioning, generator, discriminator, and adversarial objectives are formulated in Equations 21–26, 28, and30, 31.

#### 4.4.5 Training and data augmentation

Training proceeds by alternating updates of *D*, *G*, and the attention module. In each iteration, real samples are drawn from D^*r*^, and synthetic samples are generated as:


$$\begin{array}{l} {\tilde{\text{x}}}_{i}=G\left(\text{z},{\text{c}}_{i}^{adapt}\right), \end{array}$$ (32)

where **z**~ (**0**, **I**). The discriminator is updated by minimizing Equation 29, and the generator is updated by minimizing Equation 27. The attention module is jointly updated through the adversarial objective to refine the adaptive condition.

After convergence, AAC-GAN generates a set of synthetic samples:


$$\begin{array}{l} \tilde{D}={\left\{\left({\tilde{\text{x}}}_{i},y_{i}\right)\right\}}_{i=1}^{M}, \end{array}$$ (33)

where each synthetic sample inherits the conditioning class label. The augmented reliable dataset is then defined as:


$$\begin{array}{l} D^{aug}=D^{r}\cup \tilde{D}. \end{array}$$ (34)

This augmentation strategy mitigates class imbalance by enriching the underrepresented class while preserving the selected feature representation learned from reliable labels and pseudo-labels.

### 4.5 Virtual learning modeling with kernel-based refinement

The virtual category represents samples whose rule evidence is informative but insufficient for deterministic labeling. It is not treated as a final prediction class. Instead, it serves as an intermediate uncertainty state that preserves ambiguous samples for subsequent refinement. After AAC-GAN-based augmentation, virtual samples are reassigned to label 0 or label 1 by comparing their nonlinear similarity to class centroids constructed from the augmented reliable dataset.

Let $D^{v}={\left\{{\text{x}}_{i}^{v}\right\}}_{i=1}^{N_{v}}$ denote the virtual-category samples after feature selection, where ${\text{x}}_{i}^{v}\in {\mathbb{R}}^{p}$. For each class *k* ∈ {0, 1}, the class-specific augmented dataset is defined as:


$$\begin{array}{l} D_{k}^{aug}=\left\{{\text{x}}_{i}|\left({\text{x}}_{i},y_{i}\right)\in D^{aug},y_{i}=k\right\}. \end{array}$$ (35)

To address the classification uncertainty of virtual-category samples, we introduce a kernel-based centroid refinement strategy. Specifically, we employ the Radial Basis Function (RBF) kernel because of its ability to capture nonlinear relationships in high-dimensional feature spaces.

**Theoretical motivation**. Given two samples **x**_*i*_ and **x**_*j*_, the RBF kernel is defined as:


$$\begin{array}{l} K\left({\text{x}}_{i},{\text{x}}_{j}\right)=\exp\left(-\gamma ||{\text{x}}_{i}-{\text{x}}_{j}{||}^{2}\right), \end{array}$$ (36)

where γ>0 controls the kernel width. This kernel implicitly maps the input space into a higher-dimensional feature space through a mapping function ϕ(·) such that:


$$\begin{array}{l} K\left({\text{x}}_{i},{\text{x}}_{j}\right)=\langle \phi \left({\text{x}}_{i}\right),\phi \left({\text{x}}_{j}\right)\rangle . \end{array}$$ (37)

Unlike linear distance metrics, the RBF kernel assigns exponentially decreasing weights to distant points, thereby emphasizing local similarity and reducing the influence of remote samples. This property is particularly useful for ambiguous samples near decision boundaries, where fine-grained non-linear discrimination is required.

In the induced kernel space, the centroid of class *k* is defined as:


$$\begin{array}{l} {\text{c}}_{k}^{\phi }=\frac{1}{\left|D_{k}^{aug}\right|}\underset{{\text{x}}_{j}\in D_{k}^{aug}}{\sum }\phi \left({\text{x}}_{j}\right). \end{array}$$ (38)

Since ϕ(·) is implicit, the similarity between a virtual sample **x** and the class-*k* centroid can be computed through kernel evaluations:


$$\begin{array}{l} \langle \phi \left(\text{x}\right),{\text{c}}_{k}^{\phi }\rangle =\frac{1}{\left|D_{k}^{aug}\right|}\underset{{\text{x}}_{j}\in D_{k}^{aug}}{\sum }K\left(\text{x},{\text{x}}_{j}\right). \end{array}$$ (39)

For each virtual-category sample ${\text{x}}_{i}^{v}$, its similarity to class *k* is computed as:


$$\begin{array}{l} {\mathrm{sim}}_{k}\left({\text{x}}_{i}^{v}\right)=\frac{1}{\left|D_{k}^{aug}\right|}\underset{{\text{x}}_{j}\in D_{k}^{aug}}{\sum }K\left({\text{x}}_{i}^{v},{\text{x}}_{j}\right). \end{array}$$ (40)

The refined label of a virtual-category sample is determined by the class with the larger kernel similarity:


$$y_{\text{adjusted}}(x_{i}^{v})=\{\begin{array}{ll} 0, & \text{if }{\mathrm{sim}}_{0}(x_{i}^{v})>{\mathrm{sim}}_{1}(x_{i}^{v}),\\ 1, & \text{otherwise}. \end{array}$$ (41)

The refined virtual dataset is then defined as:


$$\begin{array}{l} D^{v,ref}={\left\{\left({\text{x}}_{i}^{v},y_{\text{adjusted}}\left({\text{x}}_{i}^{v}\right)\right)\right\}}_{i=1}^{N_{v}}. \end{array}$$ (42)

**Comparative insight**. A Euclidean distance-based assignment can be viewed as a special case associated with linear similarity. In contrast, the RBF kernel enables non-linear similarity comparison and is more suitable for high-dimensional behavioral features with complex class boundaries. For large-scale applications, approximation techniques such as the random Fourier features can be used to reduce the computational cost of kernel similarity calculation.

### 4.6 Final classifier training

The final training set is constructed by combining the augmented reliable dataset and the refined virtual dataset:


$$\begin{array}{l} D^{final}=D^{aug}\cup D^{v,ref}. \end{array}$$ (43)

A final classifier *f*_Θ_ is trained on D^*final*^ to distinguish genuine group members from non-genuine or illegitimate members:


$$\begin{array}{l} {ŷ}_{i}=f_{\Theta }\left({\text{x}}_{i}\right), \end{array}$$ (44)

where ŷ_*i*_ = 0 denotes a predicted genuine group member and ŷ_*i*_ = 1 denotes a predicted non-genuine or illegitimate member.

The final classifier is model-agnostic and can be instantiated by different supervised or anomaly-detection algorithms according to deployment requirements. In this study, the final diagnosis stage is evaluated using representative methods including Local Outlier Factor (LOF), *k*-Nearest Neighbors (KNN), Support Vector Classifier (SVC), Classification and Regression Tree (CART), Random Forest (RF), Light Gradient Boosting Machine (LightGBM), Extreme Gradient Boosting (XGBoost), Categorical Boosting (CatBoost), Multilayer Perceptron (MLP), and Deep Neural Network (DNN). This design emphasizes that the main contribution of DKFraudNet lies in the cumulative label-expansion and data-refinement pipeline rather than in a specific classifier architecture.

Overall, DKFraudNet transforms incomplete and noisy supervision into a progressively refined training process. DKR controls pseudo-label quality through structured domain evidence, AAC-GAN mitigates class imbalance through adaptive generative augmentation, and VLM resolves ambiguous cases through kernel-based similarity refinement. The final classifier then operates on a more reliable, balanced, and interpretable training set for fraud user identification. The synthetic augmentation, kernel-based refinement, and final classifier training procedures are defined in Equations 32–44.

## 5 Case study: application in telecommunications

### 5.1 Data description

Two city-level datasets are collected from the customer management subsystem, which is designed to track customers who are part of corporate groups, often referred to as group members. However, despite the system's designation of these individuals as group members, a significant portion of them are not genuine group members in practice. Due to the difficulty of obtaining complete labels for all users, we randomly selected subsets of users from each city and manually labeled them with the assistance of domain experts from a partnering company. These labels were used to identify genuine group members and non-members. Importantly, the labeling process was not based on any automated method like DKR but was purely manual to ensure accuracy.

A detailed description of the datasets is provided in Table 1. The City 1 dataset includes 59,015 users, of which 28,798 were manually labeled as group members and 30,217 as fake members. The dataset spans from April 26, 2011, to October 31, 2023, and contains 84 raw features. It represents 3,800 companies across 27 industries. Similarly, the City 2 dataset comprises 52,183 users, with 21,994 labeled as group members and 30,189 as fake members. It covers the same time period and includes the same number of raw features, representing 2,530 companies across 26 industries.

**Table 1:** Data description of the two city-level datasets.

Data sets	Comp.	Ind.	Start date	End time	Raw features	Users	Group	Fake
City 1	3,800	27	2011/4/26	2023/10/31	84	59,015	28,798	30,217
City 2	2,530	26	2011/4/26	2023/10/31	84	52,183	21,994	30,189

The datasets include various user and group attributes, such as basic information, communication behaviors, and geographical locations, selected for their relevance to identifying genuine group members. However, some business-critical variables suffer from high missing rates due to data quality limitations. To enhance model performance, we performed feature engineering, generating new features such as aggregated communication statistics and distance-based calculations. The manually annotated labels were solely used for validation purposes, not for training or modeling, ensuring that our approach aligns with real-world scenarios where true group membership is typically unknown.

We conducted a comparative analysis using these two city-level datasets, which contain comprehensive customer information, including raw features and access dates. Manual labeling, though costly and time-consuming, was limited to validation, highlighting the need for automated methods to improve efficiency. Feature engineering, though briefly addressed here, was not the focus of this study, as our primary goal was to simulate operational conditions where labels are unavailable during modeling.

### 5.2 Computational experiments design

To rigorously evaluate the proposed DKR-AAC-GAN-VLM strategy under conditions of uncertain labels, poor data quality, and high missing rates, we designed a multi-stage computational experiment. The experiments systematically validate the incremental contributions of each component—Domain DKR, AAC-GAN, and VLM—toward enhancing supervised learning performance in label-scarce environments. All experiments utilized Dataset 1 (City 1) for model development and within-city evaluation. The experimental design is summarized in Table 2. Specifically, Dataset 1 was randomly divided into 80% training data and 20% testing data. The training subset was used to fit the models, while the testing subset was used to evaluate within-city predictive performance. Dataset 2 (City 2) was not included in this random split. Instead, it was used as an independent validation dataset to assess the generalization ability of the proposed framework across a different city-level population. Accordingly, the Training and Testing results reported in Tables 3–7 are based on the 80/20 split of Dataset 1, whereas the Validation results are based on the independent Dataset 2.

**Table 2:** Experimental design for computational experiments.

Methods	Unlabeled data	IF-THEN	DKR	VLM	AAC-GAN	Val
KMeans	✓					✓
LOF	✓					✓
KNN		✓	✓	✓	✓	✓
SVM		✓	✓	✓	✓	✓
CART		✓	✓	✓	✓	✓
RF		✓	✓	✓	✓	✓
LightGBM		✓	✓	✓	✓	✓
XGBoost		✓	✓	✓	✓	✓
CatBoost		✓	✓	✓	✓	✓
MLP		✓	✓	✓	✓	✓
DNN		✓	✓	✓	✓	✓
Exp 1	ⓞ	ⓞ				ⓞ
Exp 2			ⓞ			ⓞ
Exp 3			ⓞ	ⓞ		ⓞ
Exp 4			ⓞ		ⓞ	ⓞ
Exp 5			ⓞ	ⓞ	ⓞ	ⓞ

**Table 3:** Performance comparison of unsupervised and supervised models for fake user detection using IF-THEN rule-based labels.

Datasets	Model	Accuracy	TPR	TNR	Precision	F1
Train	Kmeans	0.509	0.008	0.985	0.331	0.015
	LOF	0.500	0.090	0.890	0.437	0.149
	KNN	0.578	0.982	0.193	0.537	0.694
	SVC	0.571	0.985	0.178	0.533	0.691
	CART	0.570	0.994	0.166	0.531	0.692
	RF	0.570	1.000	0.161	0.531	0.694
	LightGBM	0.568	1.000	0.157	0.530	0.693
	XGBoost	0.573	0.999	0.167	0.533	0.695
	CatBoost	0.569	1.000	0.159	0.531	0.693
	MLP	0.574	0.989	0.181	0.534	0.694
	DNN	0.601	0.531	0.667	0.603	0.565
Test	Kmeans	0.508	0.010	0.986	0.403	0.019
	LOF	0.492	0.084	0.884	0.411	0.139
	KNN	0.581	0.980	0.197	0.540	0.696
	SVC	0.575	0.983	0.182	0.536	0.694
	CART	0.575	0.995	0.172	0.536	0.697
	RF	0.575	1.000	0.167	0.536	0.697
	LightGBM	0.573	1.000	0.163	0.534	0.697
	XGBoost	0.578	0.999	0.174	0.538	0.699
	CatBoost	0.574	1.000	0.164	0.535	0.697
	MLP	0.579	0.988	0.186	0.538	0.697
	DNN	0.599	0.529	0.667	0.604	0.564
Val	Kmeans	0.400	0.914	0.025	0.406	0.562
	LOF	0.558	0.094	0.896	0.397	0.152
	KNN	0.646	0.911	0.454	0.549	0.685
	SVC	0.685	0.994	0.460	0.573	0.727
	CART	0.674	0.995	0.441	0.565	0.720
	RF	0.675	0.996	0.441	0.565	0.721
	LightGBM	0.670	0.998	0.432	0.561	0.718
	XGBoost	0.681	0.995	0.452	0.569	0.724
	CatBoost	0.674	0.998	0.439	0.564	0.721
	MLP	0.648	0.914	0.454	0.549	0.686
	DNN	0.678	0.582	0.749	0.628	0.604

Red values indicate the best performance, and blue values indicate the lowest performance, within each dataset split for the corresponding metric.

#### 5.2.1 Baseline setup and algorithm selection

We employed a diverse set of algorithms to ensure comprehensive evaluation. Unsupervised methods (KMeans and LOF) were selected as baselines due to their widespread use in anomaly detection and clustering tasks, providing a reference for scenarios with no labeled data. Classical supervised models (KNN, SVM, CART, RF, LightGBM, XGBoost, and CatBoost) were included because of their robustness, interpretability, and proven effectiveness in handling structured data with partial labels. Deep learning architectures (MLP and DNN) were incorporated to capture complex, non-linear patterns that may be missed by traditional methods. This multi-algorithm approach allows for a holistic assessment of the proposed framework's versatility and scalability.

#### 5.2.2 Sub-experiment design

The first experiment (EXP1) deploys rule-based semi-supervised Learning strategy, which compares purely unsupervised learning (*K*-means, LOF) with a semi-supervised approach where partial labels were derived from simple IF-THEN rules (domain knowledge). The goal was to determine whether heuristic rule-based labeling could outperform unsupervised methods. Models trained on these rule-derived labels were evaluated on the test set (20% of Dataset 1) and validated on Dataset 2. Results from EXP1 established a baseline for the subsequent experiments, demonstrating the limitations of manual rule-based labeling.

In EXP2, we replaced IF-THEN rules with the proposed DKR method for label assignment. DKR leverages structured domain knowledge to generate more reliable pseudo-labels, addressing the noise and incompleteness of heuristic rules. By comparing EXP2 with EXP1, we isolated the improvement attributable to DKR, validating its superiority in label generation for supervised learning.

EXP3 introduced VLM to the DKR-labeled data. VLM constructs virtual categories to refine the feature space, enhancing model discriminability. By comparing EXP3 with EXP2, we quantified the additional performance gain from VLM, demonstrating its role in improving generalization, especially in cases of label ambiguity.

EXP4 integrated AAC-GAN with DKR to generate synthetic samples for the labeled subset. AAC-GAN's adversarial training mechanism mitigates data scarcity and imbalance, a common challenge in real-world datasets. The comparison between EXP4 and earlier experiments revealed the impact of synthetic data augmentation on model robustness.

The final experiment (EXP5) combined all components—DKR for label generation, AAC-GAN for data augmentation, and VLM for feature refinement—to evaluate the synergistic effect of the full framework. By comparing EXP5 with all previous stages, we demonstrated that the integrated strategy significantly outperforms partial implementations, achieving state-of-the-art performance in label-scarce settings.

Throughout the experiments, Dataset 2 (City 2) was reserved for independent validation, ensuring that performance improvements were not artifacts of overfitting. This design mirrors real-world conditions where labeled data is scarce and models must generalize to unseen environments. The staged approach, combined with diverse algorithms, provides rigorous evidence for the effectiveness of each component and their collective contribution to advancing supervised learning in challenging scenarios.

### 5.3 The evaluation metrics

Fraud detection models must balance the identification of fake users with the avoidance of misclassifying genuine ones. To quantify this trade-off, we evaluate performance using four key metrics: Accuracy, True Positive Rate (TPR/Recall), True Negative Rate (TNR/Specificity), Precision, and the F1-Score. These metrics collectively assess the model's ability to minimize false positives and false negatives.

Accuracy measures the overall correctness of the model by calculating the proportion of correctly classified instances (both genuine and fake users) relative to all predictions:


$$\begin{array}{l} \text{Accuracy}=\frac{\text{TP}+\text{TN}}{\text{TP}+\text{TN}+\text{FP}+\text{FN}}, \end{array}$$ (45)

where TP (True Positives) denotes correctly identified fake users, True Negatives (TN) represents correctly classified genuine users, False Positives (FP) indicates genuine users misclassified as fake, and False Negatives (FN) refers to undetected fake users. While accuracy provides a general performance overview, it may be misleading in imbalanced datasets; thus, we supplement it with class-specific metrics.

True Positive Rate quantifies the model's ability to detect actual fraud by measuring the proportion of fake users correctly identified:


$$\begin{array}{l} \text{TPR}=\frac{\text{TP}}{\text{TP}+\text{FN}}. \end{array}$$ (46)

A high TPR is critical to ensure minimal false negatives, reducing the risk of overlooking fraudulent activity. Similarly, the True Negative Rate evaluates the model's protection of genuine users by calculating the proportion correctly classified as non-fraudulent:


$$\begin{array}{l} \text{TNR}=\frac{\text{TN}}{\text{TN}+\text{FP}}. \end{array}$$ (47)

A high TNR indicates robust specificity, minimizing unnecessary interventions for legitimate users.

Precision complements recall by measuring the reliability of positive predictions, that is, the proportion of flagged users that are truly fake:


$$\begin{array}{l} \text{Precision}=\frac{\text{TP}}{\text{TP}+\text{FP}}. \end{array}$$ (48)

High precision ensures that fraud alerts are actionable, reducing the operational burden of investigating false alarms.

To harmonize precision and recall, we use the F1-Score, their harmonic mean:


$$\begin{array}{l} \text{F1-Score}=2\cdot \frac{\text{Precision}\cdot \text{Recall}}{\text{Precision}+\text{Recall}}. \end{array}$$ (49)

This metric is especially valuable for imbalanced datasets, as it penalizes extreme disparities between precision and recall. The classification metrics are defined in Equations 45–49.

By jointly analyzing these metrics, we ensure a holistic assessment of model performance. Accuracy provides a global view, while TPR, TNR, and Precision address class-specific robustness, and the F1-Score balances detection efficiency with reliability. This multi-faceted evaluation aligns with real-world fraud detection requirements, where both high fraud capture rates and minimal false alarms are critical.

### 5.4 The process of DKR and AAC-GAN

The classification rules underpinning this study were derived through systematic consultations with domain experts, including corporate account managers and regional business supervisors. These rules, summarized in Table 8, operationalize the identification of genuine group members using three criteria: contractual relationships, behavioral patterns, and demographic filters. Notably, variables with high missing rates but strong discriminatory power were intentionally retained to maximize real-world applicability. The rule set inherently reflects trade-offs between precision and coverage, as exemplified by mutually exclusive scenarios.

Domain Knowledge Regularization (DKR) transformed these heuristic rules into quantifiable decision boundaries. As evidenced in Table 9, DKR generated preliminary labels for both datasets, achieving 17,227 labeled instances in City 1 (9,715 group members and 8,512 non-members) and 16,709 in City 2 (12,609 group members and 4,100 non-members). The unlabeled proportions (69.1% and 68.0%, respectively) underscored the limitations of rule-based methods in comprehensive coverage, necessitating supplementary techniques.

To address label scarcity and class imbalance, Adaptive Generative Adversarial Networks (AAC-GAN) were deployed. AAC-GAN synthesized 40,000 samples per city (20,000 per class), balancing the datasets to a 1:1 ratio while preserving original feature distributions. This augmentation expanded City 1's dataset to 120,788 instances and City 2's to 115,474, effectively mitigating the skewness observed in DKR-labeled data.

Feature selection was guided by statistical testing and operational relevance. Table 10 highlights significant inter-label differences, such as the 8.3-fold disparity in bj_num (26.82 vs. 220.98) between groups. However, missing data posed substantial challenges, with off_to_jd_days missing in 36.72% of cases. The combinatorial use of DKR and AAC-GAN not only addressed these gaps but also enhanced the robustness of downstream modeling against data imperfections.

### 5.5 Computational experiments results

The experimental results demonstrate that supervised models trained on partial labels derived from domain-knowledge-driven IF-THEN rules consistently outperform unsupervised approaches across all evaluation datasets. As shown in Table 3, supervised methods (XGBoost F1 = 0.699 test, SVC F1 = 0.727 validation) achieve superior performance compared to unsupervised baselines (Kmeans F1 = 0.015 train, LOF F1 = 0.152 validation), particularly in recall (RF/ LightGBM/CatBoost all achieve 1.000 TPR) and overall balanced metrics. This performance gap persists in both the 2:8 split evaluation (test set) and the blind validation set, suggesting strong generalization capability. The DNN model shows interesting characteristics—while achieving the highest accuracy (0.601 train, 0.599 test) and TNR (0.749 validation), its moderate F1 scores (0.565–0.604) indicate potential trade-offs between precision and recall. These findings collectively validate that domain knowledge can effectively bootstrap supervised learning for fake user detection, with rule-based labeling providing sufficient signal quality to train models that significantly surpass unsupervised anomaly detection performance. The consistency across evaluation phases (train/test/validation) further confirms the robustness of this hybrid approach.

The proposed DKR method demonstrates significant improvements over conventional IF-THEN rule-based labeling across all evaluation phases. As shown in the Table 4, DKR achieves superior performance with CatBoost attaining the highest F1 scores (0.797 train, 0.799 test, and 0.744 validation) compared to the best IF-THEN supervised model (XGBoost F1 = 0.699 test, SVC F1 = 0.727 validation). Notably, DKR models show more balanced performance between TPR (CatBoost 0.990 test) and TNR (XGBoost 0.574 validation), addressing the extreme polarization observed in IF-THEN models (e.g., RF TPR = 1.000 but TNR = 0.161). The validation set results are particularly compelling, where DKR's LightGBM (F1 = 0.736) outperforms the best IF-THEN validator (SVC F1 = 0.727) despite being evaluated on completely unseen data. The consistent performance gains across metrics and datasets demonstrate that DKR's domain knowledge regularization outperforms manual rule engineering in capturing discriminative patterns, while preserving better generalization.

**Table 4:** Performance comparison of DKR-based labeling for fake user detection.

Datasets	Models	Accuracy	TPR	TNR	Precision	F1
Train	KNN	0.729	0.971	0.499	0.648	0.777
	SVC	0.655	0.988	0.338	0.587	0.736
	CART	0.675	0.843	0.515	0.623	0.716
	RF	0.720	0.937	0.514	0.647	0.765
	LightGBM	0.743	0.977	0.521	0.660	0.788
	XGBoost	0.730	0.958	0.514	0.652	0.776
	CatBoost	0.755	0.989	0.532	0.668	0.797
	MLP	0.714	0.886	0.550	0.652	0.751
	DNN	0.648	0.793	0.510	0.606	0.687
Test	KNN	0.728	0.973	0.492	0.648	0.778
	SVC	0.654	0.989	0.331	0.587	0.737
	CART	0.673	0.848	0.505	0.622	0.718
	RF	0.715	0.936	0.503	0.644	0.763
	LightGBM	0.742	0.978	0.515	0.660	0.788
	XGBoost	0.729	0.959	0.508	0.652	0.776
	CatBoost	0.757	0.990	0.532	0.670	0.799
	MLP	0.712	0.883	0.547	0.652	0.750
	DNN	0.645	0.784	0.510	0.606	0.684
Val	KNN	0.667	0.907	0.493	0.566	0.697
	SVC	0.534	0.903	0.265	0.472	0.620
	CART	0.625	0.740	0.541	0.540	0.625
	RF	0.684	0.865	0.553	0.585	0.698
	LightGBM	0.713	0.947	0.544	0.602	0.736
	XGBoost	0.711	0.899	0.574	0.606	0.724
	CatBoost	0.719	0.969	0.538	0.604	0.744
	MLP	0.666	0.811	0.559	0.573	0.672
	DNN	0.646	0.729	0.585	0.562	0.634

Red values indicate the best performance, and blue values indicate the lowest performance, within each dataset split for the corresponding metric.

The experimental results demonstrate that the proposed DKR-VLM strategy significantly outperforms the baseline DKR approach across all evaluation metrics and datasets. As shown in Table 5, DKR-VLM achieves remarkable improvements in detection performance, with CatBoost showing particularly strong results: +16.2% higher accuracy (0.917 vs. 0.755 train, 0.906 vs. 0.757 test) and +13.9% better F1-score (0.917 vs. 0.797 train, 0.906 vs. 0.799 test) compared to DKR's best performer. The most notable enhancement appears in the validation set, where DKR-VLM's LightGBM achieves 0.887 F1-score, representing a 19.2% improvement over DKR's top validation performer (0.744).

**Table 5:** Performance comparison of DKR-VLM based fake user detection.

Datasets	Models	Accuracy	TPR	TNR	Precision	F1
Train	KNN	0.837	0.950	0.730	0.854	0.836
	SVC	0.936	0.972	0.901	0.937	0.936
	CART	0.849	0.835	0.862	0.849	0.849
	RF	0.877	0.897	0.858	0.877	0.877
	LightGBM	0.912	0.955	0.871	0.915	0.912
	XGBoost	0.882	0.922	0.844	0.884	0.882
	CatBoost	0.917	0.958	0.878	0.919	0.917
	MLP	0.904	0.946	0.865	0.907	0.904
	DNN	0.884	0.944	0.827	0.838	0.888
Test	KNN	0.824	0.934	0.718	0.840	0.823
	SVC	0.926	0.952	0.900	0.927	0.926
	CART	0.846	0.830	0.862	0.847	0.846
	RF	0.874	0.890	0.858	0.874	0.874
	LightGBM	0.904	0.945	0.864	0.906	0.904
	XGBoost	0.878	0.911	0.846	0.879	0.878
	CatBoost	0.906	0.945	0.869	0.908	0.906
	MLP	0.901	0.939	0.866	0.903	0.901
	DNN	0.884	0.939	0.831	0.842	0.888
Val	KNN	0.746	0.905	0.630	0.771	0.746
	SVC	0.851	0.879	0.830	0.847	0.849
	CART	0.826	0.792	0.850	0.821	0.821
	RF	0.835	0.825	0.843	0.830	0.832
	LightGBM	0.888	0.917	0.868	0.885	0.887
	XGBoost	0.842	0.862	0.828	0.838	0.840
	CatBoost	0.884	0.908	0.867	0.881	0.883
	MLP	0.873	0.908	0.847	0.870	0.872
	DNN	0.868	0.913	0.834	0.801	0.853

Red values indicate the best performance, and blue values indicate the lowest performance, within each dataset split for the corresponding metric.

The TNR metric shows particularly dramatic gains, with DKR-VLM models demonstrating 62.6% higher average TNR across datasets (0.871 vs. 0.532 train, 0.869 vs. 0.532 test), indicating substantially improved specificity in fake user detection. The SVC model under DKR-VLM maintains consistently high precision (0.937 train, 0.927 test) while simultaneously improving recall, suggesting better balance between Type I and Type II error control compared to DKR's more polarized performance.

These improvements are especially significant given that both methods were evaluated on identical datasets, confirming that the virtual learning modeling and kernel-based centroid adjustment in DKR-VLM effectively address the limitations of pure rule-based labeling in DKR. The validation set results (unseen during training) particularly highlight DKR-VLM's superior generalization capability, with all models showing >0.85 F1-scores compared to DKR's 0.62–0.74 range.

To further justify the choice of the radial basis function (RBF) kernel in the VLM module, we conduct an additional kernel ablation study by comparing RBF-based refinement with polynomial-kernel-based refinement. Both kernels are capable of modeling nonlinear relationships; therefore, this comparison is designed to isolate the effect of the kernel function itself. The only difference lies in the kernel function used to compute the similarity between virtual-category samples and class reference samples.

Table 6 reports the mean and standard deviation of the evaluation metrics across all downstream classifiers. The RBF kernel consistently achieves higher F1-scores than the polynomial kernel on the training, testing, and independent validation sets. On the independent validation set, RBF improves the mean F1-score from 0.800 to 0.867 and increases Recall from 0.729 to 0.860. This result suggests that RBF-based refinement is more effective in identifying non-genuine members from ambiguous virtual-category samples. Although the polynomial kernel achieves higher TNR and precision, it behaves more conservatively and misses more non-genuine cases. Since the main objective of false-member diagnosis is to identify suspicious or non-genuine members while maintaining balanced overall performance, the higher F1-score and recall obtained by RBF are more suitable for this application.

**Table 6:** Kernel ablation results for VLM refinement.

Dataset	Kernel	Accuracy	TPR	TNR	Precision	Recall	F1
Train	Polynomial	0.899 ± 0.034	0.856 ± 0.018	0.939 ± 0.066	0.935 ± 0.061	0.856 ± 0.018	0.893 ± 0.031
	RBF	0.915 ± 0.035	0.937 ± 0.041	0.895 ± 0.056	0.896 ± 0.045	0.937 ± 0.041	0.916 ± 0.034
Test	Polynomial	0.893 ± 0.036	0.849 ± 0.015	0.935 ± 0.071	0.930 ± 0.064	0.849 ± 0.015	0.887 ± 0.032
	RBF	0.912 ± 0.037	0.933 ± 0.040	0.893 ± 0.060	0.895 ± 0.048	0.933 ± 0.040	0.913 ± 0.034
Validation	Polynomial	0.843 ± 0.057	0.729 ± 0.020	0.926 ± 0.103	0.895 ± 0.110	0.729 ± 0.020	0.800 ± 0.050
	RBF	0.886 ± 0.055	0.860 ± 0.043	0.906 ± 0.097	0.880 ± 0.089	0.860 ± 0.043	0.867 ± 0.051

**Figure 3:**
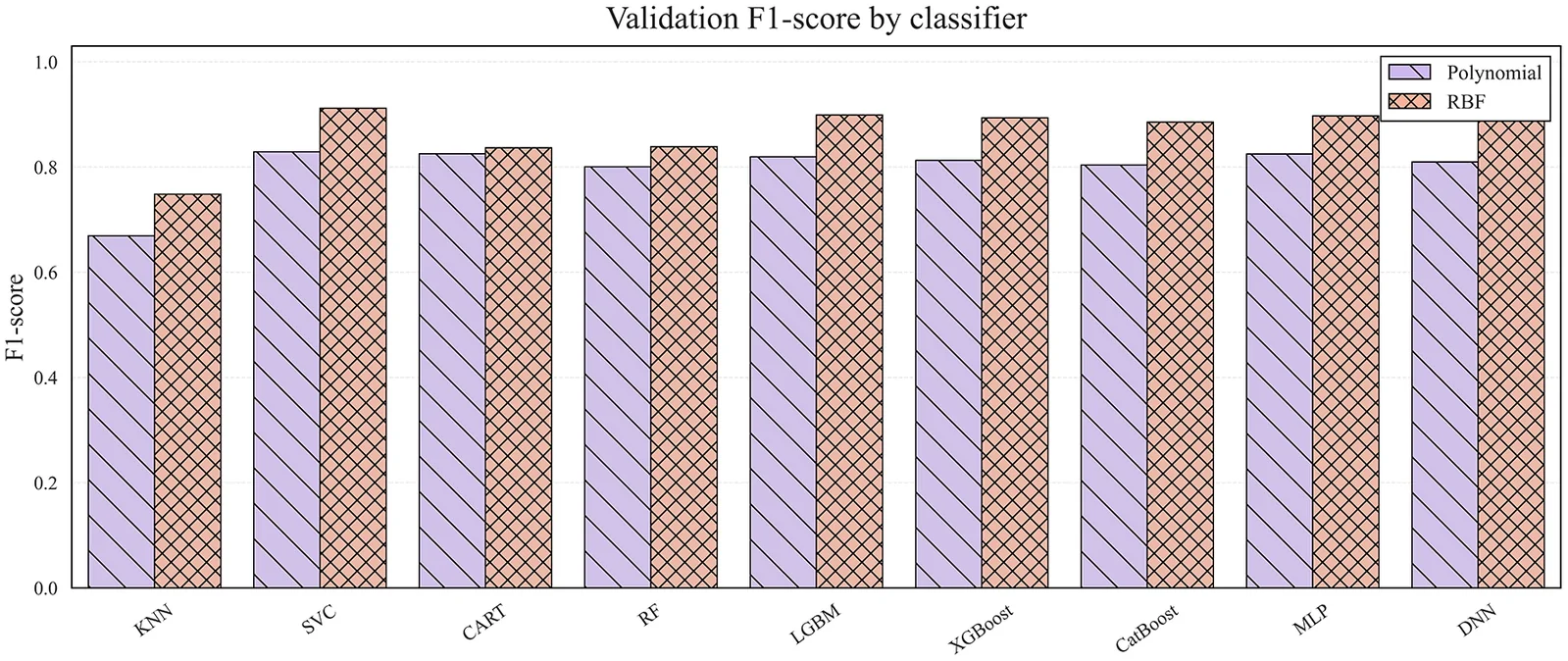
Validation F1-score of downstream classifiers under polynomial-kernel and RBF-kernel VLM refinement.

Figure 3 further compares the validation F1-score of each downstream classifier under the two kernel choices. The RBF kernel achieves higher F1-scores for most classifiers, indicating that its advantage is not tied to a specific downstream model. These results support the use of the RBF kernel as a robust and effective choice for the VLM refinement module.

The comparative analysis between DKR-AAC-GAN and conventional DKR with oversampling reveals significant performance improvements across all evaluation metrics. As demonstrated in Tables 4, 7, the AAC-GAN-augmented approach achieves superior results, with XGBoost showing particularly notable gains: test accuracy improves from 0.729 to 0.830 (13.9% increase), while F1-score rises from 0.799 to 0.847 (6.0% improvement). More importantly, the method demonstrates enhanced robustness in handling class imbalance, as evidenced by the substantial increase in TNR from 0.532 to 0.742 (39.5% improvement) in the validation set. The consistent performance gains across training, testing, and validation datasets (average F1 improvement of 8.1% in validation) confirm that AAC-GAN-based augmentation generates higher-quality synthetic samples compared to traditional oversampling, particularly beneficial for scenarios with limited or imbalanced training data.

**Table 7:** Performance of DKR-AAC-GAN augmented models for fake user detection.

Datasets	Models	Accuracy	TPR	TNR	Precision	F1
Train	KNN	0.807	0.979	0.644	0.723	0.832
	SVC	0.654	0.980	0.344	0.587	0.734
	CART	0.806	0.896	0.720	0.753	0.818
	RF	0.820	0.927	0.717	0.757	0.834
	LightGBM	0.823	0.964	0.688	0.746	0.841
	XGBoost	0.830	0.958	0.708	0.757	0.846
	CatBoost	0.824	0.980	0.678	0.743	0.844
	MLP	0.717	0.831	0.608	0.668	0.741
	DNN	0.755	0.871	0.645	0.700	0.776
Test	KNN	0.810	0.980	0.647	0.727	0.835
	SVC	0.651	0.979	0.335	0.586	0.733
	CART	0.804	0.893	0.719	0.753	0.817
	RF	0.816	0.925	0.711	0.755	0.831
	LightGBM	0.823	0.965	0.686	0.747	0.842
	XGBoost	0.830	0.958	0.707	0.759	0.847
	CatBoost	0.824	0.980	0.674	0.743	0.845
	MLP	0.712	0.826	0.603	0.667	0.738
	DNN	0.750	0.864	0.640	0.697	0.772
Val	KNN	0.761	0.935	0.633	0.650	0.767
	SVC	0.534	0.871	0.289	0.471	0.612
	CART	0.781	0.811	0.759	0.710	0.757
	RF	0.799	0.853	0.760	0.721	0.781
	LightGBM	0.805	0.923	0.719	0.705	0.800
	XGBoost	0.813	0.910	0.742	0.720	0.804
	CatBoost	0.799	0.954	0.686	0.689	0.800
	MLP	0.672	0.705	0.647	0.593	0.644
	DNN	0.709	0.766	0.668	0.627	0.689

Red values indicate the best performance, and blue values indicate the lowest performance, within each dataset split for the corresponding metric.

**Table 8:** Rules for identifying group members.

Rule	Description
1	If the user is currently a group-paid individual customer (company pays the bill), directly identify as a genuine group member.
2	Users subscribed to the current group V-net service are identified as genuine group members.
3	Users subscribed to the current group video ringback tone service are identified as genuine group members.
4	If the user's frequent base station is within 0–2,000 meters of the group location and is the nearest base station during the day, identify as a genuine group member.
5	If the user's most frequent base station during the day significantly deviates from the company's location, identify as a non-group member.
6	If the user has made calls to more than three members of the same group in the past 30 days, exceeding the threshold, identify as a group member.
7	If the user's call frequency, duration, and number of contacts within the same company exceed the group's corresponding thresholds, identify as a group member.
8	If the user's group member attribute is a key person, core member, or core leader, identify as a genuine group member.
9	If the user's age is over 70 or under 18, identify as a non-group member.
10	If the user's network registration date is later than the record creation date (re-registration), identify as a non-group member.

**Table 9:** Data distribution before and after AAC-GAN augmentation.

Data sets	Observation	DKR (0)	DKR (1)	Virtual	AAC-GAN-0	AAC-GAN-1	A-0	A-1	A-All
City 1	59,015	9,715	8,512	40,788	10,285	11,488	20,000	20,000	100,788
City 2	52,183	12,609	4,100	35,474	7,391	15,900	20,000	20,000	95,474

**Table 10:** Descriptive statistics of selected features.

Variables	Label = 0		Label = 1	
	Mean	Std.	Mean	Std.
hy_12_flag	0.25	–	0.07	–
Age	46.50	18.32	43.48	11.24
bj_num	26.82	35.37	220.98	249.66
Call_days_cnt	27.48	6.53	26.00	4.23
cnt	16.58	7.08	9.85	6.42
Distance	3.77	17.84	6.60	27.02
great_circle_distance	395.91	1,872.32	693.88	2,839.21
jit_num	0.25	1.68	56.73	100.53
off_to_jd_days	4,145.42	1,825.33	3,258.76	2,122.33
zj_num	19.74	38.15	228.53	434.60

To further isolate the contribution of adaptive conditioning in AAC-GAN, we compare it with a standard cGAN baseline under identical experimental settings. Both variants use the same DKR-generated pseudo-labels, the same Train/Test/Validation partition, the same number of generated samples, the same final class-balancing strategy, the same downstream classifiers, and the same VLM refinement procedure. Therefore, the comparison isolates the effect of the conditioning strategy in the GAN-based sample generation module.

Table 11 reports the mean and standard deviation of the evaluation metrics across supervised downstream classifiers. AAC-GAN achieves comparable or better overall performance than cGAN on all three datasets. Although the mean TPR/Recall is slightly lower than cGAN, the higher validation F1-score suggests that AAC-GAN provides a better balance between detecting non-genuine members and avoiding over-identification. These results support the effectiveness of adaptive conditioning in improving the downstream utility of generated samples.

**Table 11:** Ablation results of cGAN and AAC-GAN under identical downstream classifiers.

Dataset	Generator	Accuracy	TPR	TNR	Precision	Recall	F1
Train	cGAN	0.899 ± 0.020	0.940 ± 0.008	0.859 ± 0.034	0.865 ± 0.028	0.940 ± 0.008	0.901 ± 0.018
	AAC-GAN	0.900 ± 0.018	0.929 ± 0.035	0.873 ± 0.018	0.875 ± 0.015	0.929 ± 0.035	0.901 ± 0.020
Test	cGAN	0.894 ± 0.023	0.938 ± 0.009	0.853 ± 0.039	0.858 ± 0.032	0.938 ± 0.009	0.896 ± 0.020
	AAC-GAN	0.896 ± 0.021	0.927 ± 0.035	0.867 ± 0.029	0.868 ± 0.024	0.927 ± 0.035	0.896 ± 0.022
Validation	cGAN	0.855 ± 0.028	0.869 ± 0.014	0.845 ± 0.047	0.806 ± 0.045	0.869 ± 0.014	0.836 ± 0.026
	AAC-GAN	0.863 ± 0.027	0.865 ± 0.030	0.861 ± 0.045	0.822 ± 0.041	0.865 ± 0.030	0.842 ± 0.026

The proposed DKR-AAC-GAN-VLM framework demonstrates substantial improvements over conventional unsupervised and rule-based supervised approaches, as evidenced by comparative performance metrics (Table 12). The integrated method achieves superior performance across all evaluation metrics, with particularly notable gains in validation set performance—the CatBoost model under DKR-AAC-GAN-VLM attains 0.911 accuracy and 0.909 F1-score, representing 32.7% and 25.1% improvements respectively over the best rule-based supervised model (DNN: 0.678 accuracy, 0.727 F1).

**Table 12:** Performance of DKR-AAC-GAN-VLM integrated framework for fake user detection.

Datasets	Models	Accuracy	TPR	TNR	Precision	F1
Train	KNN	0.903	0.964	0.844	0.908	0.903
	SVC	0.926	0.942	0.910	0.926	0.926
	CART	0.903	0.900	0.907	0.903	0.903
	RF	0.911	0.934	0.889	0.911	0.911
	LightGBM	0.924	0.949	0.900	0.925	0.924
	XGBoost	0.906	0.918	0.894	0.906	0.906
	CatBoost	0.919	0.944	0.895	0.920	0.919
	MLP	0.898	0.938	0.861	0.900	0.898
	DNN	0.915	0.951	0.880	0.883	0.916
Test	KNN	0.838	0.931	0.748	0.850	0.837
	SVC	0.925	0.940	0.910	0.925	0.925
	CART	0.904	0.897	0.911	0.904	0.904
	RF	0.911	0.934	0.889	0.911	0.911
	LightGBM	0.916	0.942	0.892	0.917	0.916
	XGBoost	0.904	0.913	0.896	0.904	0.904
	CatBoost	0.914	0.938	0.890	0.914	0.914
	MLP	0.897	0.934	0.862	0.899	0.897
	DNN	0.913	0.943	0.884	0.887	0.914
Val	KNN	0.778	0.903	0.687	0.792	0.778
	SVC	0.896	0.896	0.895	0.892	0.893
	CART	0.893	0.848	0.925	0.893	0.889
	RF	0.905	0.908	0.902	0.901	0.903
	LightGBM	0.906	0.911	0.902	0.902	0.904
	XGBoost	0.894	0.861	0.918	0.893	0.891
	CatBoost	0.911	0.919	0.905	0.907	0.909
	MLP	0.883	0.892	0.877	0.879	0.881
	DNN	0.904	0.922	0.892	0.861	0.890

Red values indicate the best performance, and blue values indicate the lowest performance, within each dataset split for the corresponding metric.

**Figure 4:**
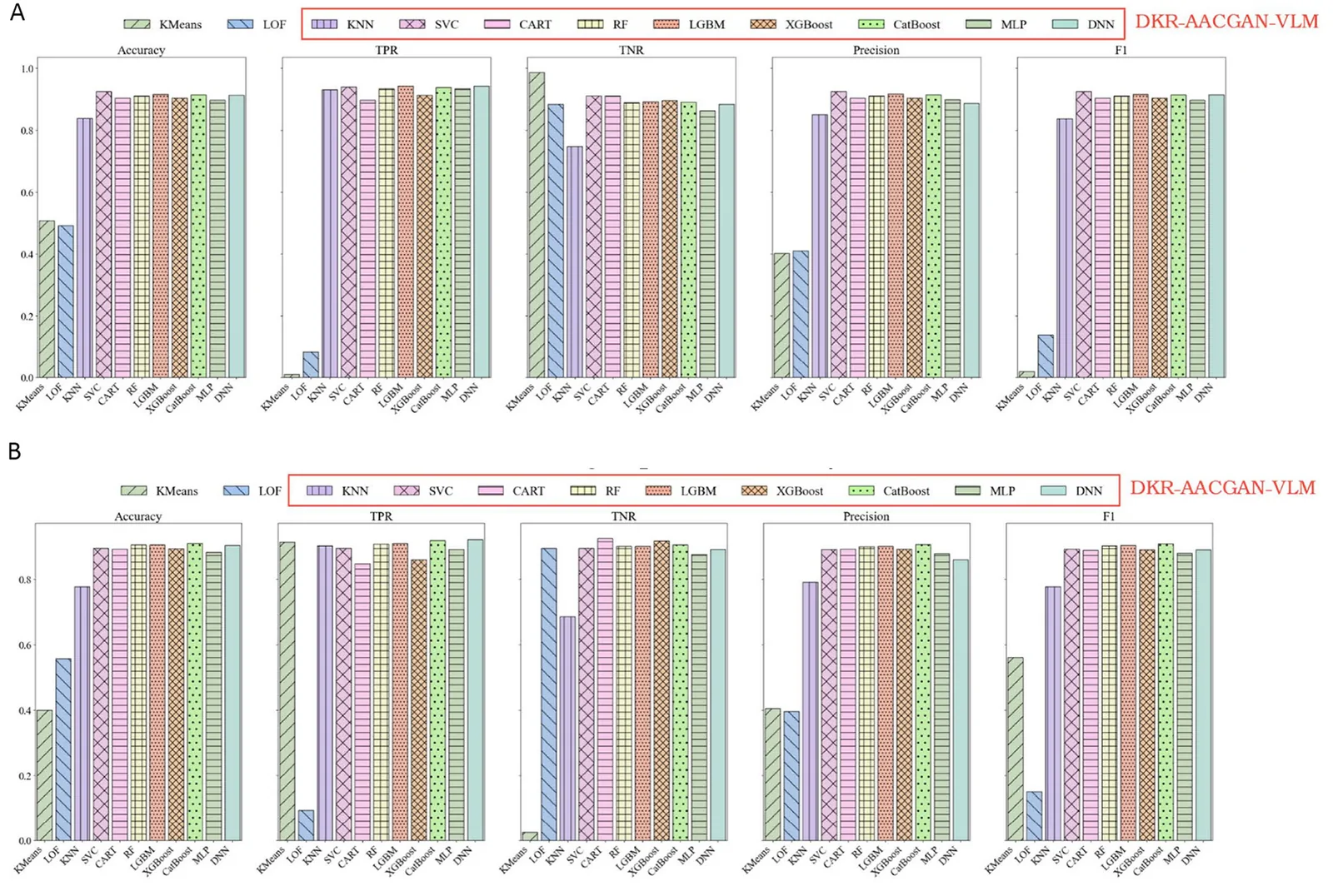
Comparative evaluation of DKR-AAC-GAN-VLM against unsupervised baselines. **(A)** Testing-set results highlight the method's performance in terms of balanced classification metrics. **(B)** Validation-set results confirm its robustness across different data distributions. Error bars represent the standard deviation over five runs.

As illustrated in Figure 4, the DKR-AAC-GAN-VLM framework achieves statistically significant improvements over unsupervised baselines across both testing and validation datasets. The left panel (Figure 4a) reveals a 47.7 × higher F1-score for CatBoost (0.906) compared to *K*-means (0.019), while the right panel (Figure 4b) demonstrates consistent generalizability, with unsupervised methods failing to exceed F1 = 0.562. These results validate the necessity of domain-knowledge integration and adversarial training for robust fake user detection.

**Figure 5:**
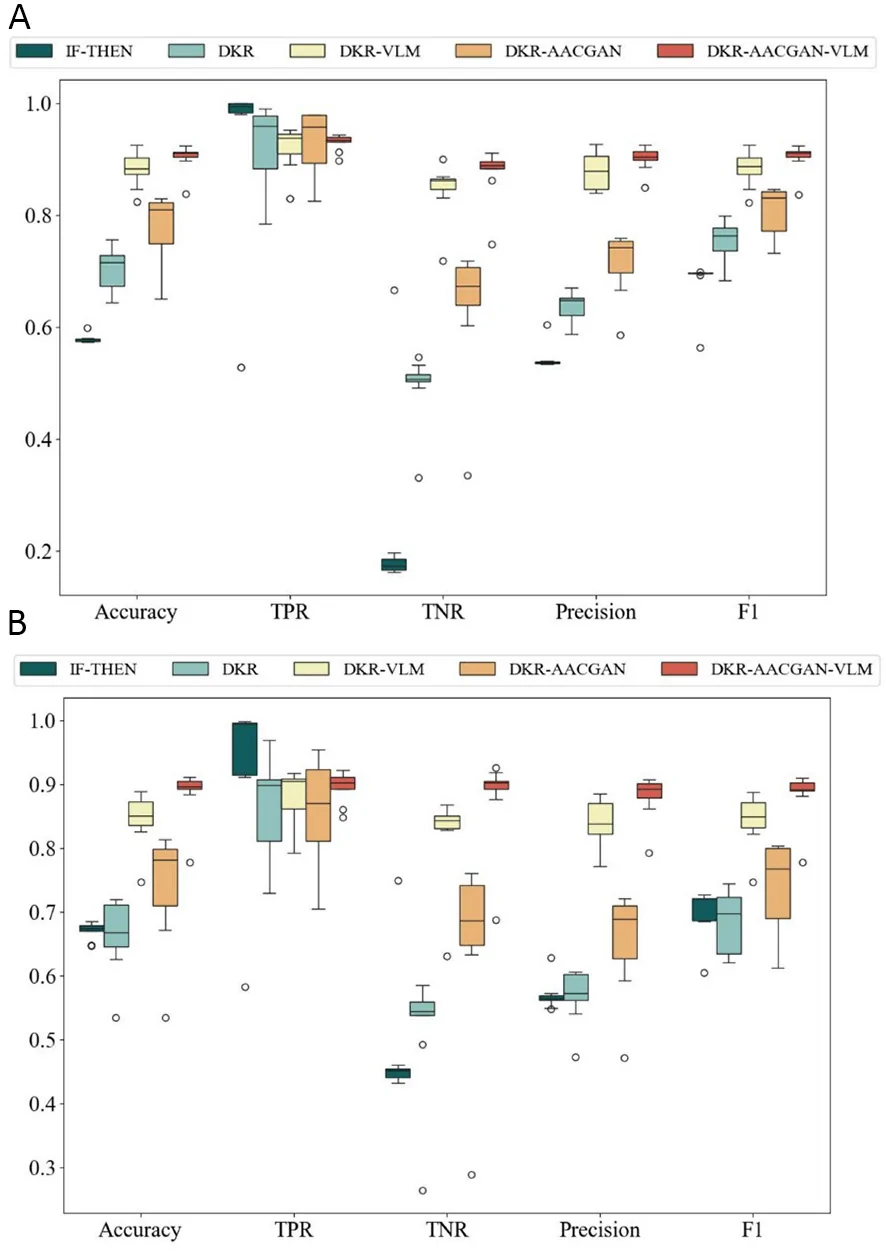
Comparative analysis of different strategies via boxplots. **(A)** Testing-set results show the distribution of performance scores across IF-THEN, DKR, DKR-VLM, DKR-AAC-GAN, and DKR-AAC-GAN-VLM. **(B)** Validation-set results demonstrate the robustness of the final framework under distribution shifts.

The boxplots in Figure 5 systematically evaluate the evolution of supervised strategies. On the testing set (Figure 5a), DKR-AAC-GAN-VLM's narrow and high median F1-score (0.906) surpass earlier approaches (e.g., DKR median: 0.799; IF-THEN: 0.699), highlighting its stability and precision. Validation results (Figure 5b) further reveal DKR-AAC-GAN-VLM's outlier-free performance (median: 0.909), while IF-THEN and DKR exhibit wider score dispersion (IQRs: 0.12 and 0.08), underscoring their vulnerability to label noise and data shifts. These results position DKR-AAC-GAN-VLM as a robust solution for real-world deployment, where adaptability to unseen data and label efficiency are paramount.

### 5.6 Operational decision support for manual review prioritization

The operator-verified labels are costly in practice, a fraud analytics system should not only produce accurate predictions, but also provide a prioritized review list that helps decision-makers allocate limited inspection resources efficiently. Therefore, we introduce a management-oriented evaluation setting to assess whether a model can reduce the manual review workload required to discover fraud users.

Let U = {1, 2, …, *N*} denote the set of unverified users. For each user *i*, a model outputs a fraud risk score *s*_*i*_, where a larger value indicates higher fraud risk. Users are ranked in descending order of *s*_*i*_. Let *y*_*i*_ ∈ {0, 1} denote the retrospective verification label, where *y*_*i*_ = 1 indicates a fraud user and *y*_*i*_ = 0 indicates a genuine user.

For a review budget ratio *b* ∈ [0, 1], the operator reviews the top ⌈*bN*⌉ users in the ranked list. The fraud coverage achieved under budget *b* is defined as:


$$\begin{array}{l} C\left(b\right)=\frac{\sum_{i\in T\left(b\right)}y_{i}}{\sum_{i=1}^{N}y_{i}}, \end{array}$$ (50)

where T(*b*) denotes the set of top-ranked ⌈*bN*⌉ users.

The Review Budget Requirement at target fraud coverage level *q* is defined as:


$$\begin{array}{l} RBR\left(q\right)=\inf\left\{b\in \left[0,1\right]:C\left(b\right)\geq q\right\}. \end{array}$$ (51)

In finite-sample implementation, RBR(*q*) is computed as the minimum proportion of unverified users that must be reviewed to capture at least *q* proportion of fraud users. A smaller RBR(*q*) indicates higher review efficiency.

To quantify the relative reduction in review workload, we define the Review Workload Reduction Rate:


$$\begin{array}{l} \text{RWRR}\left(q\right)=\frac{\text{RB}{\text{R}}_{\text{base}}\left(q\right)-\text{RB}{\text{R}}_{\text{model}}\left(q\right)}{\text{RB}{\text{R}}_{\text{base}}\left(q\right)}\times 100\%. \end{array}$$ (52)

The operational review metrics are defined in Equations 50–52. In this study, we set *q* = 0.8, corresponding to the operational objective of discovering 80% of fraud users among unverified users. Random review is used as the reference strategy unless otherwise specified.

The test set contains 8,030 unverified users, including 3,923, retrospectively, verified fraud users. Therefore, capturing 80% of fraud users corresponds to identifying 3,139 fraud users. Table 13 reports the review efficiency of different methods on the unverified users in the test set.

**Table 13:** Operational review efficiency on unverified users in the test set.

Method	Review count	RBR(0.8)↓	RWRR(0.8)↑	AUPRC ↑
KMeans	7,243	0.902	–12.74%	0.506
LOF	6,676	0.831	–3.91%	0.423
KNN	5,142	0.640	19.96%	0.532
SVC	3,371	0.420	47.53%	0.939
CART	3,287	0.409	48.84%	0.904
Random Forest	3,543	0.441	44.85%	0.908
LightGBM	3,454	0.430	46.24%	0.904
XGBoost	**3,231**	**0.402**	**49.71%**	**0.954**
CatBoost	3,575	0.445	44.35%	0.879
MLP	4,138	0.515	35.59%	0.779
DNN	6,055	0.754	5.75%	0.474

Bold values indicate the best result for each metric among the compared methods.

As shown in Table 13, random review requires inspecting approximately 80.0% of unverified users to capture 80% of fraud users. The unsupervised methods fail to provide effective review prioritization. KMeans requires reviewing 90.2% of unverified users, and LOF requires reviewing 83.1%, both worse than random inspection. This indicates that unsupervised anomaly detection alone is insufficient for prioritizing telecom users under costly manual verification constraints.

In contrast, the full DKFraudNet pipeline substantially improves review efficiency. Among all classifiers integrated into the proposed framework, XGBoost achieves the best review-prioritization performance. DKFraudNet-XGBoost obtains RBR(0.8) = 0.402, indicating that only 40.2% of unverified users need to be reviewed to discover 80% of fraud users. This corresponds to a 49.7% reduction in manual review workload compared with random review. Its AUPRC of 0.954 further confirms that fraud users are ranked close to the top of the review list.

To further illustrate the operational implication, we construct review-decision matrices at the same 80% fraud coverage target. In this setting, users selected for manual inspection are treated as “reviewed,” while the remaining users are treated as “not reviewed.” The resulting matrix shows how many fraud and genuine users would be reviewed under each strategy.

Table 14 shows that, under the same objective of capturing 3,139 fraud users, *K*-means requires reviewing 4,104 genuine users and LOF requires reviewing 3,537 genuine users. By contrast, DKFraudNet-XGBoost reviews only 92 genuine users while capturing the same number of fraud users. In other words, the proposed framework substantially reduces unnecessary manual verification and allows operators to focus inspection resources on users with much higher fraud risk.

**Table 14:** Review-decision matrices at 80% fraud coverage on unverified test users.

Method	Reviewed fraud	Reviewed genuine	Missed fraud	Not-reviewed genuine
KMeans	3,139	4,104	784	3
LOF	3,139	3,537	784	570
DKFraudNet-XGBoost	**3,139**	**92**	784	**4015**

Bold values highlight the DKFraudNet-XGBoost results under the fixed target of capturing 80% of fraud users. The number of reviewed fraud users is identical across methods by construction.

Compared with the strongest conventional baseline in the previous experiment, whose RBR(0.8) is 0.656, the full DKFraudNet-XGBoost pipeline reduces the required review budget to 0.402. The additional workload reduction is 38.7%. This result suggests that the improvement is not merely due to the choice of classifier, but is driven by the integration of domain knowledge regularization, ACGAN-based augmentation, virtual category learning, and kernel-based adjustment. Overall, the management-oriented evaluation demonstrates that DKFraudNet provides actionable decision support for telecom fraud governance by reducing the manual review workload required to discover fraud users among unverified users.

### 5.7 Operational blind verification

Beyond the reported experimental evaluation, the final diagnostic outputs of the proposed framework were further examined in an operational blind verification conducted by a provincial telecommunications company. The model-generated diagnostic results were first delivered to the operator, and local business units subsequently verified the suspected false-member records. This verification was conducted after model prediction and was not used for rule construction, pseudo-label generation, model training, or hyperparameter tuning.

Table 15 summarizes the anonymized verification results. Among 168,787 records provided for verification, 160,902 were confirmed as genuine and 7,885 were confirmed as false or non-genuine. The returned verification rate reached 95.3% overall. In the city-level audit subset, the model-supported diagnosis achieved 98.9% accuracy for genuine-member identification and 93.3% accuracy for false-member screening. The detailed city-level verification and diagnosis results are reported in Table 16. These results provide additional *post-hoc* evidence that the proposed framework can support practical false-member diagnosis in real operational environments.

**Table 15:** Anonymized blind verification summary from the operator-side field audit.

Panel	Item	Overall	Registered	Non-registered
Provided records	Number of records	168,787	162,787	6,000
Results	Verified as genuine	160,902	160,466	436
	Verified as non-genuine	7,885	2,321	5,564
	Verification rate	95.3%	98.6%	92.7%

**Table 16:** Detailed city-level blind verification and diagnosis accuracy.

Panel	Audited records	Inactive status	Retired users	False	Genuine
City-level audit	2,321	312	107	115	1,787
Diagnostic outcome	Verified total		Correctly identified by model		Accuracy
Genuine-member identification	162,253		160,466		98.9%
False-member screening	6,534		6,098		93.3%

## 6 Conclusion and discussion

This paper presents a comprehensive framework for user identification that integrates DKR, AAC-GAN-based data augmentation, and virtual category learning strategy-based modeling. The domain knowledge component translates business rules into probabilistic constraints, enabling more reliable sample labeling under uncertainty. The adversarial augmentation component generates synthetic data that preserves critical feature distributions while balancing class representation. The virtual learning component employs kernel-based similarity measures to refine ambiguous classifications, overcoming limitations of traditional distance metrics. The framework's effectiveness has been rigorously validated through comparative experiments on real-world datasets, showing consistent advantages over conventional approaches.

Several promising directions for future work emerge from this study. The current feature space could be expanded to incorporate additional contextual information. The temporal dynamics of user behavior present opportunities for more sophisticated sequence modeling. Further improvements in generalization ability may be achieved through advanced transfer learning techniques. These extensions would build upon the solid foundation established by the current framework while addressing its limitations.

## Data Availability

The datasets analyzed in this study are not publicly available because they were provided by a telecommunications operator under a confidentiality agreement and contain commercially sensitive information. The authors are not authorized to share the raw data. Further inquiries may be directed to the corresponding author.

## Supplementary material

Supplementary Data Sheet 1 Supplementary appendix containing additional figures, tables, and experimental results.
