# Supplementary material for: DKFraudNet: a knowledge-guided adversarial learning framework for fraud user detection
Source: Front Big Data. 2026 Aug 12;9:1887075. doi: 10.3389/fdata.2026.1887075 (PMC13506494; doi:10.3389/fdata.2026.1887075)
Supplement: Supplementary file 1 [file Data_Sheet_1.PDF]

Supplementary Material

1 DATASET DESCRIPTION AND FEATURE SUMMARY

This supplementary section provides a detailed description of the dataset used in the computational experiments. The dataset consists of user-level and group-level operational records provided by a telecommunications operator. All records were anonymized before analysis, and no personally identifiable information was used in the modeling process. Tables S1 and S2 summarize the original variables used in this study.

The original variables span seven dimensions: demographic attributes, group affiliation information, behavioral indicators, financial metrics, tariff-related variables, temporal records, and geographic information. Demographic variables describe user characteristics; group-level variables capture group identifiers, membership structure, and organizational attributes; behavioral variables summarize call activities, payment behavior, and service usage; financial variables reflect revenue contribution and payment amount; tariff-related variables describe plan types and fee structures; temporal variables record key business events such as registration, activation, and update dates; and geographic variables characterize the spatial relationship between users and their affiliated groups. The category abbreviations used in the tables are defined as follows: Demo = demographic, GrpInfo = group information, Behav = behavioral, Fin = financial, Tariff = tariff-related, Temp = temporal, and Geo = geographic.

The dataset contains heterogeneous missingness patterns. Some variables exhibit high missing rates; for example, several tariff-related variables and short-code call indicators have missing rates above 80%. Temporal variables, such as user registration dates and tariff plan effective dates, also contain missing values. In contrast, several core behavioral and tenure-related indicators are relatively complete. Nevertheless, the combination of behavioral, financial, temporal, and spatial information provides a rich basis for characterizing user–group consistency and analyzing the authenticity of corporate group members.

In addition to the original variables, we constructed several derived features to better characterize temporal consistency, spatial proximity, and group-member interaction intensity. Table S3 reports the definitions and missing rates of these generated features. The temporal features measure intervals between key business events, including user registration, tariff plan activation, group registration, and the latest information update. The spatial features quantify the distance between a user's frequent daytime location and the registered location of the affiliated group. Threshold-based variables were further generated based on domain knowledge to provide reference benchmarks for evaluating the interaction intensity between users and their affiliated groups.

Figure S1 and S2 illustrates the distributions of selected features grouped by the target variable, denoted as `Actual_label`. The categorical feature `hy_12_flag` is visualized using a count plot, whereas continuous variables are shown using histograms with kernel density estimation. The observed distributional differences between classes suggest that the selected variables capture behavioral, financial, temporal, and spatial patterns associated with corporate group-member authenticity.

Table S1. Field Descriptions (Part 1)

No.	Column Name	Description	Category	Miss.Rate
1	county_id	County ID code where the user is located	Demo	34.335%
2	group_id	Group ID code where the user belongs	GrpInfo	4.401%
3	group_seq	Group registration ID code	GrpInfo	34.335%
4	iden_no	ID number of the group's registration document	GrpInfo	34.377%
5	plan_id	User tariff code	Tariff	34.335%
6	plan_id_v	Group tariff code	Tariff	84.357%
7	plan_name1	Group tariff name	Tariff	34.513%
8	plan_name_v	Name of the user's current base tariff plan	Tariff	84.357%
9	vpmn_cust_id	Group network code	GrpInfo	69.456%
10	addr_flag	Document address annotated during group registration	GrpInfo	99.412%
12	cust_acct_name	Member type annotated within the group	GrpInfo	99.545%
13	group_level_cn	Level of the user's entity group	GrpInfo	4.342%
14	group_manager_id	ID code of the customer manager registering user information	GrpInfo	34.444%
15	group_number_attr_name	User's rank attribute within the group	GrpInfo	4.677%
16	hy_12_flag	Code of the industry (out of 12 major industries) the group belongs to	GrpInfo	34.335%
17	iden_id	Type of the group's registration document	GrpInfo	34.335%
18	if_0	Whether the user is a zero-usage account	Behav	0.000%
20	if_call	Whether the user had calls with 3+ group members in the last 30 days	Behav	1.698%
21	if_m2m	Whether the user is a non-voice user (e.g., IoT, landline)	Behav	34.335%
22	if_photo	Group level as determined by the telecom group	GrpInfo	34.335%
23	if_sho	Whether the user is part of a group VPMN/short-code network	Behav	65.665%
24	if_spcl	Whether the user has subscribed to video ringback tones	Behav	1.698%
25	if_szg	Whether the group is a provincial-level directly managed group	GrpInfo	34.335%
26	if_szgkey	Whether the group is a branch of a provincial-level directly managed group	GrpInfo	34.336%
27	if_tf	Whether the user is a unified payment user	Behav	1.698%
28	if_v	Whether the user is a group VPMN user	Behav	65.665%
29	plan_zifei	User tariff tier	Tariff	0.100%
30	trade_type	Industry code	GrpInfo	4.342%
31	plan_zifei1	Current base tariff plan fee	Tariff	34.513%
32	create_date	User registration date	Temp	10.670%
33	create_date_jd	Registration date of the user's entity group	Temp	34.335%
34	create_date_off	Effective date of the current base tariff plan	Temp	36.722%
35	done_date	Last information update date	Temp	34.335%
36	net_create_date	User's network activation date	Temp	0.000%
37	age	Age	Demo	2.375%
38	amount	Average monthly payment frequency	Behav	18.166%
39	arpu_x	Average monthly ARPU	Fin	2.436%
40	attr_3	Number of core members in the user's group	GrpInfo	4.395%

Table S2. Field Descriptions (Part 2)

No.	Column Name	Description	Category	Miss.Rate
41	attr_4	Number of contacts in the user's group	GrpInfo	4.395%
42	attr_5	Number of key members in the user's group	GrpInfo	34.376%
43	attr_all	Total number of key members in the group	GrpInfo	4.395%
44	bj_count	Number of incoming calls in the last 6 months	Behav	11.016%
45	bj_num	Number of unique incoming callers in the last 6 months	Behav	9.444%
46	bj_yiwang_num	Number of incoming calls from non-network numbers in the last 6 months	Behav	9.444%
47	call_days	Number of days with calls in the last 6 months	Behav	89.191%
48	call_days_cnt	Average monthly days with data usage	Behav	2.187%
49	cnt	Average monthly billed call duration	Behav	9.444%
50	fee_group_num	Total number of revenue-generating ABC groups in the locality	GrpInfo	34.336%
51	gjrjt_calldura_m	Call duration with three types of group members in the last 6 months	Behav	96.406%
52	gjrjt_count	Number of calls with three types of group members in the last 6 months	Behav	96.406%
53	gjrjt_num	Number of unique callers among three types of group members in the last 6 months	Behav	42.788%
54	group_num	Total number of group members	GrpInfo	4.395%
55	info_fee	User's average monthly revenue contribution (in CNY)	Fin	4.355%
56	jf_cnt	Average monthly payment amount	Fin	18.166%
57	jit_num	Number of unique callers among group members in the last 6 months	Behav	9.444%
58	jt_calldura_m	Call duration with group members in the last 6 months	Behav	65.185%
59	jt_count	Number of calls with group members in the last 6 months	Behav	65.185%
60	latitude	Latitude of the user's group location	Geo	8.892%
61	longitude	Longitude of the user's group location	Geo	8.892%
62	mou_bhd	Average monthly billed call duration	Behav	2.382%
63	nojt_calldura_m	Call duration with non-group members in the last 6 months	Behav	10.097%
64	nojt_count	Number of calls with non-group members in the last 6 months	Behav	10.097%
65	nojt_num	Number of unique callers among non-group members in the last 6 months	Behav	9.444%
66	sho_call_count	Average monthly number of group VPMN short-code calls in the last 6 months	Behav	72.434%
67	sort_fee	User's average monthly revenue contribution ranking	Fin	17.909%
68	user_online	User's network tenure (in days)	Temp	0.000%
69	vw_call_count	Average monthly number of group VPMN calls in the last 6 months	Behav	72.434%
70	work_latitude	Latitude of the user's daytime frequent location	Geo	13.712%
71	work_longitude	Longitude of the user's daytime frequent location	Geo	13.712%
72	yy_ct	Average monthly voice saturation	Behav	2.382%
73	zj_count	Number of outgoing calls in the last 6 months	Behav	13.244%
74	zj_num	Number of unique outgoing callers in the last 6 months	Behav	9.444%
75	zj_yiwang_num	Number of outgoing calls to non-network numbers in the last 6 months	Behav	9.444%

Table S3. Generated Features and Threshold Parameters

Feature Name	Description	Type	Miss.Rate
days_since_last_update	Days between the current base tariff plan's effective date and the last update date	Numerical	76.739%
days_since_signup	Days between registration and the last update date	Numerical	34.335%
off_to_jd_days	Days between the current base tariff plan's effective date and group registration date	Numerical	36.722%
distance	Distance between the user's daytime frequent location and the group location	Numerical	17.479%
great_circle_distance	Great-circle distance between frequent location and group location	Numerical	17.479%
distance_threth	Threshold for judging distance between frequent location and group location	Numerical	36.039%
jit_num_threth	Threshold for the number of unique callers among group members in the last 6 months	Numerical	71.429%
jt_calldura_m_threth	Threshold for call duration with group members in the last 6 months	Numerical	71.271%
jt_count_threth	Threshold for the number of calls with group members in the last 6 months	Numerical	71.329%

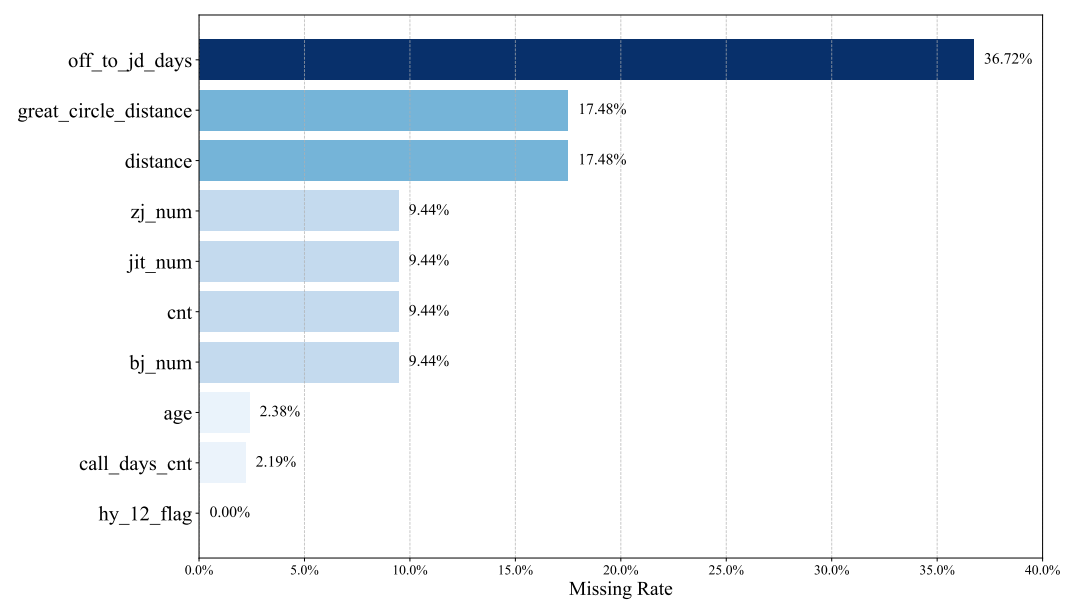

Figure S1: Missing rate of selected features for modeling

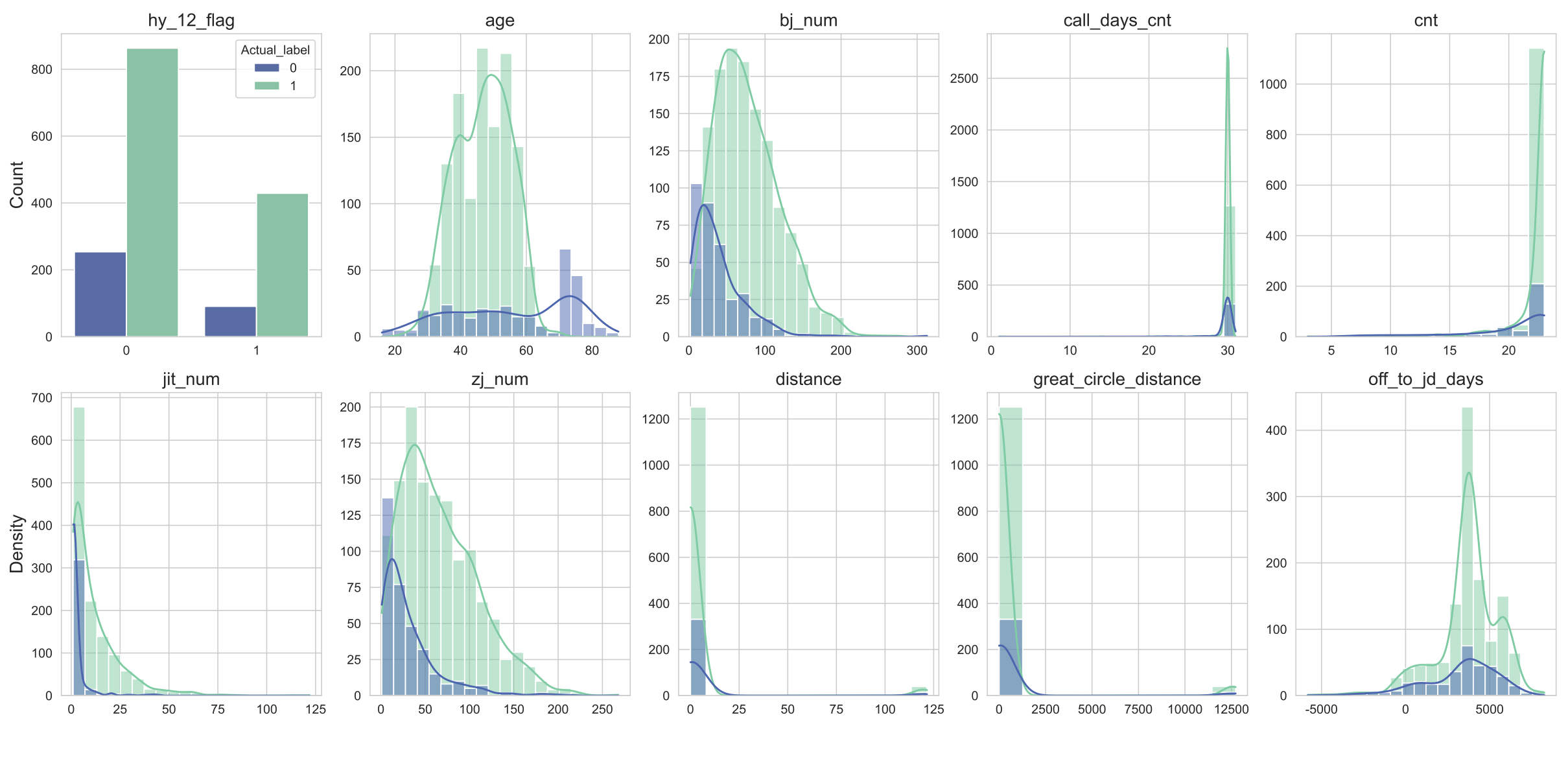

Figure S2: Distributions of selected features grouped by the target variable `Actual_label`.
